# Microbiome Associated with *Polypedilum* sp. (Diptera; Chironomidae), a Midge Adapted to an Extremely Acidic Environment

**DOI:** 10.1264/jsme2.ME24090

**Published:** 2025-06-21

**Authors:** Eita Nakanishi, Richard Cornette, Sachiko Shimura, Takahiro Kikawada

**Affiliations:** 1 Department of Integrated Biosciences, Graduate School of Frontier Sciences, The University of Tokyo, Kashiwa, Chiba 277–8562, Japan; 2 Institute of Agrobiological Sciences, National Agriculture and Food Research Organization (NARO), Tsukuba, Ibaraki 305–0851, Japan

**Keywords:** chironomid, acid tolerance, microbiome, rRNA gene amplicon sequencing

## Abstract

Chironomids (Diptera; Chironomidae), non-biting midges, are a highly diverse family of holometabolous insects, many of which are known for their tolerance to extreme environmental conditions, such as desiccation, pollution, and high acidity. The contribution of microbial symbionts to these adaptations was recently suggested. Therefore, we herein exami­ned the microbiome associated with the larvae of the undescribed acid-tolerant chironomid species, *Polypedilum* sp., which inhabits the Yukawa River (Gunma, Japan), an environment that is characterized by an extremely low pH (≤2) and high concentrations of heavy metal ions (including arsenic). Amplicon sequencing of the 16S rRNA gene revealed a distinct larval microbiome with a lower alpha diversity value and more enriched and specific bacterial taxa than the surrounding river water and detritus. Full-length 16S rRNA gene sequencing using nanopore long-read technology identified several previously undescribed operational taxonomic units (OTUs), among which OTU_*Bacillaceae*_Yukawa was consistently present in larvae reared in the laboratory for more than 4 months, suggesting persistent, possibly vertically transmitted, symbiosis. An inferred pathway ana­lysis suggested the contribution of the larval microbiome to host nutritional physiology. The possibly acid-sensitive OTU_*Bacillaceae*_Yukawa localized to midgut segments, indicating internal pH-buffered niches for microbial survival. These results provide novel insights into the ecology of acid-tolerant chironomids and lay the groundwork for further examinations of holobiont-based stress tolerance.

Chironomids (Diptera; Chironomidae), commonly known as non-biting midges, are a diverse group of holometabolous insects comprising at least 7,290 described species (Cornette *et al.*, 2023). The majority of chironomids complete larval development in aqueous environments, feeding on a broad range of organic matter, such as detritus and other dead invertebrates (Berg, 1995). Many chironomid species exhibit tolerance to extreme conditions, including pollution, desiccation, elevated concentrations of heavy metal ions, and high acidity (Hinton, 1960; Sugg *et al.*, 1983; McLachlan *et al.*, 1995; Kawai *et al.*, 2019). In addition to genomic adaptations in chironomids, their relationship with specific microorganisms may contribute to their tolerance to extreme conditions. Recent studies that investigated the adaptation of chironomids to extreme habitats and targeted both the host and associated microbes, referred to as the holobiont, suggested that microorganisms affected the tolerant phenotype of the host (Rosenberg *et al.*, 2007; Simon *et al.*, 2019; Sela and Halpern, 2022). Chironomids inhabiting polluted environments were found to harbor endogenous bacterial communities that detoxify a range of toxic substances, including heavy metal ions, and chemicals, such as toluene (Senderovich and Halpern, 2013; Sela and Halpern, 2019; Sela *et al.*, 2021). Nevertheless, the symbiotic relationship between local microbes and host chironomids in the context of adaptation to extreme habitats has yet to be clarified.

Our interest focuses on chironomid holobionts that have adapted to inhabit extremely acidic environments (pH of ~2 or lower). Japan is a volcanic archipelago comprising a number of acidic environments, including hot springs, mountain streams, and caldera lakes. Some of the chironomid species that inhabit these acidic environments in Japan have been documented (Jernelöv *et al.*, 1981; Doi *et al.*, 2019; Kawai *et al.*, 2019; Fujii *et al.*, 2021). Of these, *Chironomus acerbiphilus*, which inhabits Lake Katanuma, a caldera lake in Miyagi, Japan, is the most acid-tolerant species, with a pH tolerance of 2.0 (Kawai *et al.*, 2019). A comparative ana­lysis of the microbiome of acid-tolerant chironomids, including *C. acerbiphilus*, and non-tolerant species using laboratory-reared strains revealed that acid-tolerant *Chironomus* larvae were associated with microbiomes that had low alpha diversity indexes and featured certain species-specific microorganisms (Fujii *et al.*, 2023).

The main focus of the present study was the ecosystem of the Yukawa River, which flows through Kusatsu hot spring in Gunma, Japan. The Yukawa River is characterized by an extremely acidic pH (approximately 2.0), which is similar to the acidity observed in Lake Katanuma, the habitat of *C. acerbiphilus*. However, the concentration of heavy metal ions in the Yukawa River is at least three-fold higher than that in Lake Katanuma (Sato, 1995; Kikawada *et al.*, 2014). Furthermore, the concentration of arsenic in the Yukawa River may be several tens of times higher than that in Lake Katanuma (Noguchi and Nakagawa, 1968; Kikawada *et al.*, 2009), making the river an even more extreme environment. These conditions are clearly unsuitable for the survival of fish, crustaceans, or macrophytes. However, we discovered an undescribed chironomid species belonging to the genus *Polypedilum* (*Polypedilum* sp.), which undergoes approximately 1 month of larval development, as the dominant macroinvertebrate species in the Yukawa River. Therefore, we hypothesized that *Polypedilum* sp. may exhibit extreme acid tolerance at a similar level to that of *C. acerbiphilus* as well as superior metal ion resistance to that of *C. acerbiphilus*.

In the present study, we conducted a comparative ana­lysis of the microbiome composition of the acid-tolerant *Polypedilum* sp. with that of water from the Yukawa River and detritus collected directly from the river bottom. The results obtained revealed the presence of specific microorganisms that were enriched in the larvae of acid-tolerant *Polypedilum* sp. chironomids.

## Materials and Methods

### Collection of insects and environmental samples

**Fig. 1.**
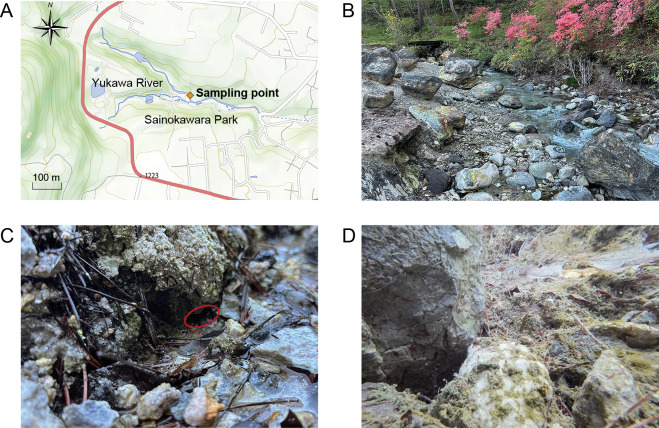
Location of the sampling site along the Yukawa River in Sainokawara Park. (A) Sampling point for water, detritus, *Polypedilum* sp. egg masses, and larvae along the Yukawa River in Sainokawara Park. The pH and temperature at this point were 2.11 and 13.3°C, respectively. The map of the sampling site was reproduced from the GSI Maps Vector published by the Geospatial Information Authority of Japan. (B) View of the Yukawa River. (C) The point at which egg masses were sampled. The red circle indicates where egg masses were attached. (D) *Polypedilum* sp. larvae colonizing the river bottom (small red bloodworms).

Sampling was conducted on May 17, 2024 in the Yukawa River‍ ‍within Sainokawara Park (Gunma, Japan; 36°37′27.1″ N, 138°35′23.5″ E; altitude 1,180 m; Fig. 1A and B). The pH and temperature at the sampling points were assessed using the compact pH meter model pH-33B (Horiba). The egg masses and larvae of acid-tolerant *Polypedilum* sp. (one specimen per sample) were collected using a sterile dropper. Egg masses embedded in a gelatinous matrix and laid on rocks above the water level near the stream were collected (Fig. 1C). Larvae were collected from colonies in the water (Fig. 1D). To remove surface-associated microbes, larvae were washed by vortexing five times in sterile water immediately after sampling, as described in previous studies (Halpern *et al.*, 2007; Sela *et al.*, 2021). Samples of detritus (400–450‍ ‍mg each) were collected from the river bottom at locations where larvae were present using a sterile syringe. As samples of river water, 1 L of water was filtered through a 0.22-μm Sterivex GP filter unit (Merck Millipore). After filtration, 2‍ ‍mL of DNA/RNA Shield (Zymo Research) was added to the membrane filter to preserve DNA. Upon transport to the laboratory, the DNA/RNA Shield reagent was collected from the filter unit by centrifugation. Operating within a laminar flow cabinet, the internal filter membrane was retrieved by disassembling the filter unit. To minimize external contamination, river water was collected using sterile tubes with tube pumping equipment. Videos of swimming larvae of *Polypedilum* sp. can be accessed online (https://doi.org/10.6084/m9.figshare.27237672.v1).

### Rearing of acid-tolerant chironomids

The larvae of acid-tolerant *Polypedilum* sp. were transported to‍ ‍our laboratory along with river water from their natural habitat and they were subsequently reared under controlled laboratory conditions. Larvae were maintained in a plastic rearing tank (35.4×24.2×12‍ ‍cm) with river water at 27–28°C under a 13-h light:11-h dark cycle and were fed finely ground autoclaved fish food (Tetrafin^®^; Tetra Werke). Importantly, larvae were continuously reared for more than 4 months without replacing the original river water (pH ≤2). Since the life cycle of *Polypedilum* sp. spans approximately one month, this rearing period corresponds to at least 4 generations. DNA was isolated from reared larvae and used as a “laboratory-reared larval sample”. The pH of water samples was measured, and 50‍ ‍mL of rearing water was filtered through a 0.22-μm Sterivex^TM^ GP filter unit (Merck Millipore) to collect DNA on the filter membrane. To minimize sampling bias, water was collected simultaneously from three distinct locations near the corners of the tank.

### Dissection of larvae

Chironomid larvae consist of a head capsule with a rigid cuticular structure and a body composed of 12 segments (see the right panel of Fig. 6C). Segments 1 to 3 make up the thorax, while segments 4 to 12 form the abdomen. A digestive tract runs from the mouth to the anus, consisting of three distinct regions: the foregut (in the thorax, segments 1–3), the midgut (in the anterior abdomen, segments 4–8 or 9), and the hindgut (in the posterior abdomen, segments 9 or 10–12). This anatomical arrangement is somewhat analogous to the human digestive system: the foregut corresponds to the esophagus and stomach, the midgut functions similar to the small intestine, and the hindgut is equivalent to the large intestine and rectum. To investigate the spatial distribution of the gut microbiota within the larval body, surface-washed larvae were dissected using fine scissors into 4 blocks, each with 3 segments. DNA was then extracted from each segmental block individually for a microbial ana­lysis.

### Extraction of total DNA, including microbial DNA

Total DNA was isolated using a NucleoSpin^®^ Soil kit (Macherey-Nagel) and a Micro Smash^TM^ MS-100 cell disruptor (Tomy Seiko) for homogenization (2,000‍ ‍rpm, 15‍ ‍min). Lysis Buffer SL2 and Enhancer SX were used for cell lysis.

Collected egg masses, surface-washed larvae, dissected larval segments, and river sediment (detritus) samples were transferred into MN bead tubes containing ceramic beads for disruption. DNA extraction was then performed according to the manufacturer’s protocol for the NucleoSpin^®^ Soil kit.

Regarding the extraction of DNA from water, one-half of the membrane filtered with water samples, along with 1‍ ‍mL of the collected DNA/RNA Shield solution, was carefully transferred into‍ ‍an MN bead tube containing ceramic beads, and DNA was then extracted according to the manufacturer’s protocol for the NucleoSpin^®^ Soil kit.

### Short-read sequencing for the 16S rRNA gene ana­lysis

Samples of DNA extracted from egg masses (*n*=3), larvae (*n*=3), detritus (*n*=3), and river water (*n*=3) were used for a 16S rRNA gene ana­lysis. ZymoBIOMICS^TM^ Microbial Community DNA Standard (Zymo Research) was employed as a technical quality control (*n*=1) to assess the reliability of the workflow from library preparation to the 16S rRNA gene sequencing ana­lysis (*i.e.*, as a ‘mock community’). Target sequences were amplified using Takara Ex Premier^TM^ DNA Polymerase Dye plus (Takara Bio). The target V3–V4 region of the 16S rRNA gene was amplified using the 16S rRNA gene universal primers 341F (5′-CCTACGGGGNGGCWGCAG-3′) and 805R (5′-GACTACHVGGGGTATCTAATCC-3′). PCR was performed under the following conditions: denaturation at 94°C for 1‍ ‍min; 30 cycles of denaturation at 98°C for 1‍ ‍min, annealing at 55°C for 40‍ ‍s, and extension at 68°C for 90 s; followed by a final extension at 72°C for 2‍ ‍min. The resulting amplicons were purified using AMPure XP beads (Beckman Coulter) and used for library construction and sequencing with 2×300 bp of the V3–V4 region of the 16S rRNA gene on a MiSeq platform (Illumina) by Bioengineering Laboratory.

### Bioinformatics ana­lysis based on the sequence of the 16S rRNA gene V3–V4 region

To identify amplicon sequence variants (ASVs), QIIME2 (version 2023.9) (Bolyen *et al.*, 2019) with the dada2-plugin (q2-dada2; version 2023.9) (Callahan *et al.*, 2016) was used to trim and filter raw data, denoise and merge paired-end reads, and remove chimeric sequences. Trimming resulted in a maximum sequence of 280 nt for forward reads and 200 nt for reverse reads, resulting in an approximately 430-bp V3–V4 region. Our Github page shows the actual code used in the present study (https://github.com/Kikawada-Lab-UT-NARO/Microbiota_Acid_Chironimid).

ASVs were then subjected to taxonomic annotation using the Silva 138 SSU Ref NR99 database with the qiime feature-classifier plugin (Glöckner *et al.*, 2017; Bokulich *et al.*, 2018). Sequences derived from chloroplast 16S rRNA genes were excluded from the ASV table. The accuracy of the phylogenetic classification and the percentage of each phylogeny were evaluated based on the mock community. A rarefaction ana­lysis was performed to assess sampling depths using the qiime diversity alpha-rarefaction command (Fig. S1). Alpha diversity, Chao 1, Shannon entropy, and beta diversity values were evaluated using the qiime diversity core-metrics-phylogenetic command at a sampling depth of 10,349 reads, resulting in weighted UniFrac distances. Bar plots were generated using the qiime taxa barplot command. Each processed file was visualized using QIIME2 view (https://view.qiime2.org). Principal coordinate ana­lysis (PCoA) data were plotted using the‍ ‍qiime2R (version 0.99.6) tool (https://github.com/jbisanz/qiime2R). The linear discriminant ana­lysis effect size (LefSe) (version 1.1.01) (Segata *et al.*, 2011) was used to compare microbiomes between sample groups. Data were analyzed at the order level and revealed significant differences in abundance between larvae and other samples. The normalization value was set at 1 million.

### Inference of pathway abundance in the microbiome based on 16S rRNA gene sequencing

Order-level pathway abundance was predicted using QIIME2 with the q2-picrust2 plugin (version 2024.5_0) (https://github.com/picrust/q2-picrust2; compatible with PICRUSt2 2.5.2) (Douglas *et al.*, 2020). The count table and sequence data for ASVs derived from short-read sequencing of the V3–V4 region of the 16S rRNA gene were input to this pipeline. The results obtained were annotated as inferred pathways based on the classification provided in the MetaCyc database (https://metacyc.org). To visualize differences in the abundance of the inferred pathways between larvae and‍ ‍river water samples, pathway data were analyzed using the ggpicrust2 package (version 1.7.3) (Yang *et al.*, 2023) including edgeR (version 4.2.2) (Robinson *et al.*, 2010). The false discovery rate was measured using the Benjamini & Hochberg method (Benjamini and Hochberg, 1995) at a setting of <0.05; results were then shown via a heatmap generated using the “*pathway_heatmap*” function.

### Long-read sequencing of full-length 16S rRNA genes to classify bacterial species

Full-length 16S rRNA gene sequencing was performed using the same DNA templates from larvae and water that were used for the V3–V4 short-read ana­lysis. The target DNA encompassing the V1–V9 regions was amplified using the primers 27F (5′-AGAGTTTGATYMTGGCTCAG-3′) and 1492R (5′-TACGGYTACCTTGTTACGACTT-3′). PCR was performed under the following conditions: denaturation at 94°C for 1‍ ‍min; 30 cycles of denaturation at 98°C for 1‍ ‍min, annealing at 55°C for 1‍ ‍min 30‍ ‍s, and extension at 68°C for 90 s; followed by a final extension at 72°C for 2‍ ‍min. The sequencing library was constructed using Native Barcoding kit 96 V14 SQK-NBD114.96 (Oxford Nanopore Technologies), Blunt/TA Ligase Master Mix (New England Biolabs), NEBNext FFPE Repair Mix (New England Biolabs), the NEBNext Ultra II End repair/dA-tailing Module (New England Biolabs), and the NEBNext Quick Ligation Module (New England Biolabs) according to the manufacturers’ instructions. The library was purified using AMPure XP beads, loaded onto a FLO-FLG114 SQK-LSK114 (R10.4.1) flow cell, and then sequenced using a MinION sequencer equipped with a Flongle adapter (Oxford Nanopore Technologies) and MinKNOW software (v24.02.8).

### Analyses of full-length 16S rRNA genes

FASTQ files were generated by duplex basecalling and demultiplexing using Dorado (https://github.com/nanoporetech/dorado; version 0.7.2). Sequenced long reads were filtered, leaving only 1,000 to 2,000 bp and Q9 or better reads using NanoFilt (version 2.8.0) (De Coster *et al.*, 2018). NanoCLUST (version v1.0dev) (Rodríguez-Pérez *et al.*, 2021) was used to taxonomically classify full-length 16S rRNA gene sequences at species-level resolution. Relative abundance was verified using parameters with –min_cluster_size of 100 and 25. 16S rRNA gene assemblies were validated using known sequences from a mock community, which allowed for the adjustment of ana­lysis parameters. The mock community reference sequence was downloaded from the Zymo Research website (https://s3.amazonaws.com/zymo-files/BioPool/ZymoBIOMICS.STD.refseq.v2.zip). The mock community reference and assembly sequences were aligned using Multiple Alignment program based on Fast Fourier Transform (**MAFFT**) version 7.520 (Katoh and Standley, 2013) (Option: --auto). Jalview (version 2.11.4.1) was used to visualize multiple sequence alignment (MSA) (Waterhouse *et al.*, 2009). The 16S rRNA gene sequences obtained were compared with those in the National Center for Biotechnology Information (NCBI) 16S rRNA sequence database (https://ftp.ncbi.nlm.nih.gov/blast/db/v5/16S_ribosomal_RNA.tar.gz) to identify the closest relatives of each strain. Sequences annotated with 16S rRNA genes from chloroplasts were excluded. A BLASTn ana­lysis using sequence data for ASVs defined by the V3–V4 region as queries was performed to confirm the adequacy of microbial classifications based on the full-length 16S rRNA gene sequences obtained.

### Generation of phylogenetic trees for selected taxa

The bacterial 16S Ribosomal RNA RefSeq Targeted Loci Projects for *Acidithiobacillales*, *Bacillaceae*, and *Eubacteriales* were downloaded from NCBI (download dates: July 27, 2024, July 27, 2024, and August 7, 2024, respectively). MAFFT version 7.520 was used for MSA of estimated full-length 16S rRNA gene sequences (Option: --auto). Phylogenetic trees were generated using the IQ-TREE2 (version 2.2.5) program (Minh *et al.*, 2020). The appropriate substitution model was selected using ModelFinderPlus based on the Bayesian Information Criterion (BIC). The ultrafast bootstrap feature was repeated 1,000 times (Option: -m MFP -bb 1000 -alrt 1000 -nt 4). The best-fit models TN+F+R2 (*Acidithiobacillales*), SYM+I+R9 (*Bacillaceae*), and TVMe+I+R6 (*Eubacteriales*) were selected according to BIC. Interactive Tree of Life (iTOL) (Letunic and Bork, 2024) was employed to visualize phylogenetic trees. The phylogenetic tree for *Acidithiobacillales* was constructed using *Thermithiobacillus* spp. as an outgroup based on a previous study (Moya-Beltrán *et al.*, 2021).

## Results

### Analysis of the V3–V4 region of 16S rRNA genes

The surface-washed larvae and egg masses of acid-tolerant chironomid *Polypedilum* sp., along with samples of river water and detritus from their habitat, were collected from the Yukawa River in Kusatsu hot spring (Gunma, Japan). Total DNA, including DNA from microbes associated with chironomid samples, was extracted from these samples on-site. The resulting DNA samples were used as templates for a short-read sequencing ana­lysis of amplicons of the V3–V4 region of 16S rRNA genes. The number of reads obtained for each sample ranged between 33,873 and 73,355. After sequence filtering, chloroplast reads were removed, leaving between 10,349 and 22,841 non-chimeric reads (Table S1).

The reliability of the workflow from library preparation to 16S rRNA gene sequencing was evaluated by establishing whether the results of the same workflow for the mock community corresponded with the anticipated taxonomic classification and composition profiles. The mock community consisted of a mixture of eight distinct types of bacterial DNA present in a specific ratio. Theoretical bacterial composition profiles were compared with profiles deduced from sequencing data for the mock community. The resulting taxonomic annotation and composition profiles were consistent with the expected theoretical composition profiles (Fig. S2), indicating that biases related to PCR amplification in the 16S rRNA gene sequencing ana­lysis in the present study were limited.

### Microbiome alpha and beta diversity values

**Fig. 2.**
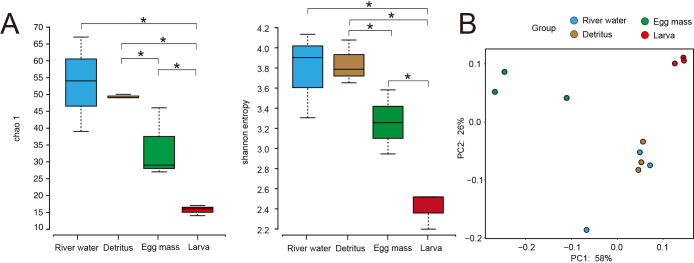
Comparison of alpha and beta diversities of the 16S rRNA gene composition associated with river water, detritus, egg mass, and larval microbiomes. (A) Comparison of samples based on the alpha diversity index using the Kruskal-Wallis test. Asterisks indicate differences with *P*<0.05. The left panel shows the Chao 1 index, and the right panel shows the Shannon entropy index, which indicate microbial richness and microbial diversity, respectively. (B) A PCoA plot based on weighted UniFrac distances for each sample.

Alpha and beta diversity ana­lyses were conducted to identify microbial diversity indicators in acid-tolerant *Polypedilum* sp. and the Yukawa River environment. To assess diversity according to microbial richness, the Chao 1 index was calculated, returning mean values of 53.3 for river water, 49.3 for detritus, 34.0 for egg masses, and 15.7 for larvae (Fig. 2A, left panel). The Shannon entropy index was calculated to assess diversity in terms of microbial abundance and evenness, and returned values of 3.78 for river water, 3.84 for detritus, 3.26 for egg masses, and 2.41 for larvae (Fig. 2A, right panel). An ana­lysis of these values using the Kruskal-Wallis test revealed that the Chao 1 and Shannon entropy indices both demonstrated significantly reduced alpha diversity values among larvae. The Shannon entropy index also revealed significant differences between detritus and egg masses. To further investigate differences between the microbiomes of acid-tolerant *Polypedilum* sp. and their habitat, beta diversity was evaluated using PCoA plots based on weighted UniFrac distances for the larval microbiome (Fig. 2B). Detritus and egg masses both exhibited some degree of inter-sample variability, whereas minimal inter-sample variation was observed for larvae and river water. Egg mass samples were located far from the other samples in the PCoA plot.

### Microbiome composition in the Yukawa River and larvae of acid-tolerant *Polypedilum* sp.

**Fig. 3.**
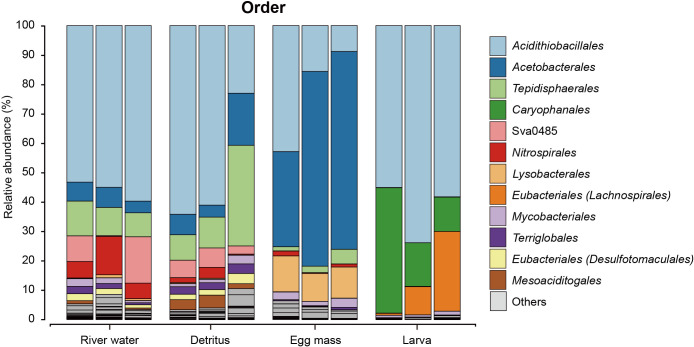
Relative abundance of 16S rRNA gene sequences associated with microbiomes of larvae, eggs, and environmental samples. A taxonomy bar plot for samples of river water, detritus, eggs, and larvae (*n*=3) at the order level. The Y axis represents relative frequency. Colors indicate taxonomic annotation.

To classify the microbiomes associated with larvae, egg masses, detritus, and river water, bar plots were constructed to visualize the relative abundance of specific taxa (Fig. 3 and S3). We conducted a comparative ana­lysis at the order level to identify the distinctive features of the microbiomes. *Acidithiobacillales* was identified in all samples at a minimum level of 8.8%. *Caryophanales* and *Eubacteriales* were detected primarily in larvae, but also to a lesser extent in egg mass samples (Fig. S4). The specific percentages of microbial taxa in each sample were as follows, with the mean of three samples shown in parentheses. In river water, *Acidithiobacillales*, the dominant order, represented 53.3–59.7% (56.0%) of the sampled material, whereas *Tepidisphaerales* accounted for 8.2–11.9% (9.9%). The relative abundance of the Sva0485 clade was in the range of 0.3–15.8% (8.2%), with *Nitrospirales* at 5.3–13.1% (8.0%) and *Rhodospirillales* at 4.0–6.8% (5.7%). In detritus, the relative abundance of *Acidithiobacillales* was 23.0–64.2% (49.5%), *Tepidisphaerales* 8.7–34.3% (17.8%), *Rhodospirillales* 4.1–17.7% (9.6%), and Sva0485 2.8–6.6% (5.1%). The relative abundance of bacteria in egg masses was 32.3–67.3% (55.3%) for *Rhodospirillales*, 8.8–42.9% (22.5%) for *Acidithiobacillales*, and 9.5–12.2% (10.8%) for *Lysobacterales*. In larvae, the relative abundance of *Acidithiobacillales* was 55.1–73.9% (62.4%), whereas those of *Caryophanales* and *Eubacteriales* were 11.7–42.7% (23.1%) and 0.6–27.1% (12.5%), respectively. The mean relative abundance of the remaining taxa in larvae was <1.0%.

### Identification of bacteria enriched in acid-tolerant larvae

**Fig. 4.**
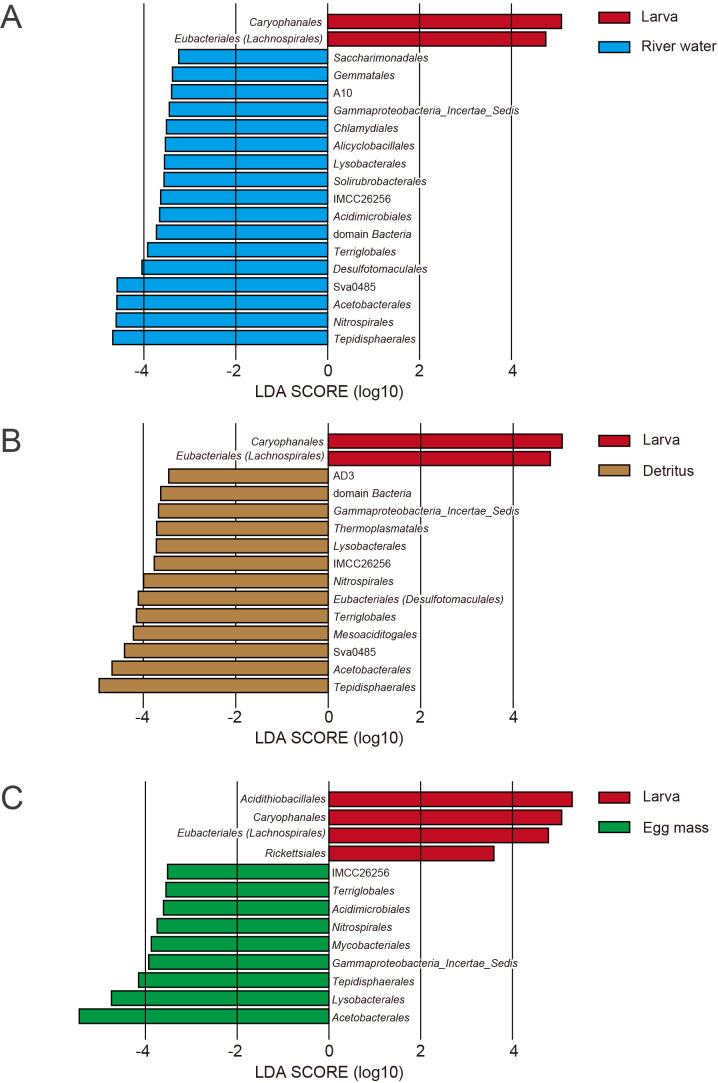
Taxonomic differences in 16S rRNA gene sequences between larvae and other sample types. Bars (red: larvae; blue: river water; brown: detritus; green: egg masses) represent Linear Discriminant Analysis scores (>2.0) for unique taxa in each set of samples. (A) Comparison of larvae and river water. (B) Comparison of larvae and detritus. (C) Comparison of larvae and egg masses.

To identify significant between-sample differences in the representation of bacterial strains, microbial differential abundance was assessed using LEfSe. In comparisons of samples of river water and larvae, *Caryophanales* and *Eubacteriales* were more abundant in larvae, whereas 17 taxa, including *Tepidisphaerales*, *Nitrospirales*, and *Rhodospirillales*, were more prevalent in river water (Fig. 4A). A comparison of samples of detritus and larvae revealed that *Caryophanales* and *Eubacteriales* were more‍ ‍abundant in larvae, whereas 13 taxa, including *Tepidisphaerales*, *Rhodospirillales*, and Sva0485, were more abundant in detritus (Fig. 4B). A comparison between samples of larvae and egg masses showed that larvae were‍ ‍enriched with *Acidithiobacillus*, *Caryophanales*, *Eubacteriales*, and *Rickettsiales*, whereas egg masses were enriched with nine taxa, including *Rhodospirillales*, *Lysobacterales*, and *Tepidisphaerales* (Fig. 4C). In comparisons with the other sample types, acid-tolerant larvae were frequently enriched in *Caryophanales* and *Eubacteriales* (Fig. 4).

### Prediction of metabolic pathway abundance in the microbiome of acid-tolerant larvae

**Fig. 5.**
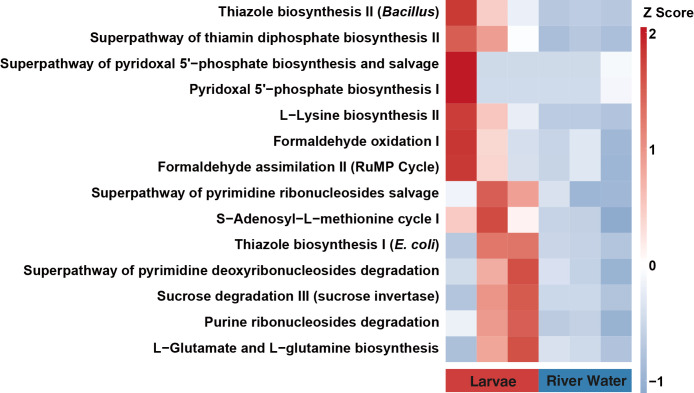
Inferred abundance of various metabolic pathways based on the 16S rRNA gene community associated with microbiomes of larvae and Yukawa River water. The characteristics of the abundance of metabolic pathways in the larval microbiome were inferred using PiCRUSt2. Metabolic pathways are listed on the left, and each column corresponds to one sample (chironomid larva [single individual] or water sample). The specific color of each row indicates the Z score for enriched or depleted pathways.

To evaluate the potential roles of the microbiomes, the metabolic pathways of microbiomes that were enriched in larvae samples were inferred using the PICRUSt2 pipeline based on the abundance of 16S rRNA gene short-reads. A total of 337 pathways were inferred to be enriched in the bacterial communities of larvae and water samples. Notably, a comparison of larvae and water samples showed that 114 pathways were enriched (Fig. S5), of which 14 were more abundant in the larval microbiome, as shown by the heatmap in Fig. 5. These 14 pathways included thiazole biosynthesis II (*Bacillus*), the superpathway of thiamine diphosphate biosynthesis II, L-lysine biosynthesis II, S-adenosyl-L-methionine cycle I, and purine ribonucleosides degradation. However, it is important to note that a large degree of variability was observed between individual larvae.

### Species-level classification of microbiomes associated with acid-tolerant larvae and water exami­ned using long-read 16S rRNA gene sequencing

Among the ASVs in the V3–V4 region of 16S rRNA genes in larvae samples, only *Acidithiobacillus ferrooxidans* was annotated at the species level with a relative abundance >0.5%. No other taxa were identified at the species level (Fig. S6). However, almost full-length sequencing of the 16S rRNA gene, comprising the V1–V9 regions, enabled the subsequent classification of bacteria to the species level (Johnson *et al.*, 2019; Zhang *et al.*, 2023). Therefore, the nanopore long-read sequencing of amplicons of full-length 16S rRNA genes was conducted using microbial DNA derived from acid-tolerant larvae that was also used for short-read amplicon sequencing (Table S2). The assembly of full-length 16S rRNA genes using DNA from the mock community ensured that the gene sequences from larval microorganisms were accurate, with the exception of gaps at the 5′ and 3′ ends.

The results of the mock community assembly revealed a minimum similarity of 99.66% (Fig. S7 and S8, Table S3). The assembled almost full-length 16S rRNA gene sequences of larval samples ranged between 1,445 and 1,491 bp in length. The use of the NanoCLUST pipeline enabled the sequences of six operational taxonomic units (OTUs) to be reconstructed, with only one OTU exhibiting a sequence similarity >98.65%. Furthermore, the sequence of this OTU showed a >99% match with ASVs in the V3–V4 region of a member of the same taxon.

Taxonomic classification using BLAST in the NanoCLUST pipeline and phylogenetic tree construction were performed to identify the most abundant bacterial genera (defined as those with an average relative abundance ≥1.0%) and OTUs enriched in larvae. Although Hackmann’s (2025) thresholds are not yet widely adopted, they represent the most recent criteria and were applied in this study. Phylogenetic ana­lyses are shown in Fig. S9, S10, and S11, and classification results are summarized in Table 1.

### Changes in larval and water microbiomes due to differences in rearing environments between the natural habitat and laboratory conditions

**Fig. 6.**
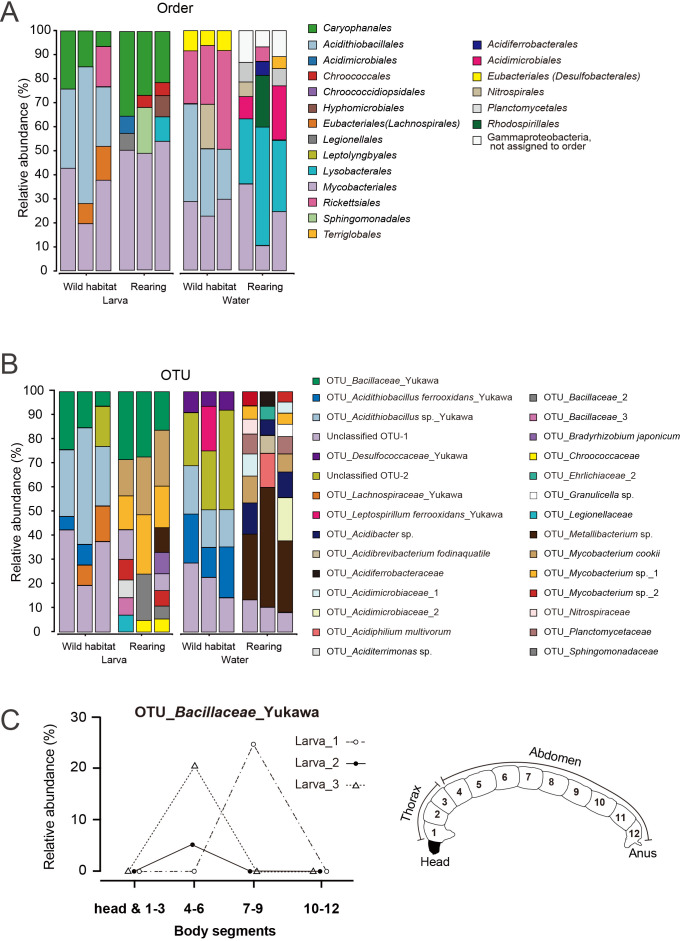
Relative abundance of 16S rRNA gene sequences associated with microbiomes in the wild habitat or under laboratory rearing conditions. The 16S rRNA gene community compositions of larvae and river water were compared between laboratory and wild habitat conditions using a long-read ana­lysis of full-length 16S rRNA genes. (A) An order-level taxonomic bar plot for larval samples (*n*=3) collected from the wild Yukawa River habitat or under laboratory rearing conditions. The Y axis represents relative abundance. Colors indicate taxonomic annotation. (B) The same taxonomic bar plot annotated at the smallest OTU level. (C) Relative abundance of OTU_*Bacillaceae*_Yukawa in different segments of the larval body. A simplified schematic illustration of the larval morphology on the right shows the positions of the different segments of the thorax and abdomen indicated on the X axis of the graph to the left.

The effects of the rearing environment of chironomids on the associated microbiome were exami­ned by comparing the microbiome composition of DNA extracted from lab-reared larvae with that of water and DNA collected on-site from larvae and water in the habitat. The DNA samples obtained were used as templates for the nanopore sequencing of full-length 16S rRNA genes (Fig. 6A, B, and S12). Even among chironomid larvae transferred from the natural environment of the Yukawa River to the laboratory rearing environment, *Caryophanales* and *Mycobacteriales* were commonly present at the order level (Fig. 6A, Table S4). Comparisons at the species level (*i.e.*, OTU) revealed that the relative abundance of OTU_*Bacillaceae*_Yukawa remained in the range 6.8–30.4% under both conditions (Fig. 6B, Table S4). In contrast, under laboratory rearing conditions, neither *Acidithiobacillus* (including both OTU_*Acidithiobacillus ferrooxidans*_Yukawa and OTU_*Acidithiobacillus* sp._Yukawa) nor OTU_*Lachnospiraceae*_Yukawa were detected in the larval microbiome (*i.e.*, below the detection limit). A comparison of the microbiome composition of water samples (*n*=3) between laboratory and natural habitat conditions showed that none of the bacteria found in river water were detected in rearing water in the laboratory, except for Unclassified_OTU-1 (Fig. 6B, Table S5).

To clarify the specific localization of OTU_*Bacillaceae*_Yukawa within larvae, we compared the relative abundance of bacteria in different body segments using nanopore sequencing. OTU_*Bacillaceae*_Yukawa strain was only detected in the middle portion of the larval body, such as segments 4–6 or 7–9 (Fig. 6C and S13).

## Discussion

The present study investigated the microbiome associated with an acid-tolerant *Polypedilum* sp. that inhabits a harsh environment characterized by a combination of high acidity and elevated concentrations of heavy metal ions. Since research on the microbiome of acid-tolerant chironomids is limited to data obtained from laboratory-passaged strains (Fujii *et al.*, 2023), the primary aim of the present study was to assess the chironomid microbiome in the natural habitat environment in order to obtain insights into interactions between the acid-tolerant chironomid and bacteria that may be related to acid tolerance. 16S rRNA gene sequencing was conducted to elucidate the microbiome composition of acid-tolerant *Polypedilum* sp. and its habitat environment and revealed the enrichment of unique, previously unknown bacterial OTUs in *Polypedilum* sp. larvae (Table 1).

### The Yukawa River microbiome may sustain the growth of acid-tolerant *Polypedilum* sp.

It is reasonable to posit that the microbiome of the riverine environment exami­ned in the present study may also exhibit acid tolerance. Previous studies on the microbiome of the Yukawa River at Sainokawara Park identified several unique algae and bacteria using classical microscopic methods (Takayanagi *et al.*, 1986; Nagashima, 1995). We herein performed the high-throughput amplicon sequencing of 16S rRNA genes, which is an effective approach for elucidating the composition and phylogeny of microorganisms in environmental samples with culture-independent mole­cular techniques.

Previous studies on microorganisms that inhabit the Yukawa River showed that the following photosynthetic unicellular red algae exhibited acid tolerance: *Cyanidium caldarium* (Tilden) Geitler, *Galdieria sulphuraria* (Galdieri) Merola, *Pinnularia braunii* var. *amphicephala*, *Pinnularia* sp., and *Chroococcidiopsis thermalis* var. *nipponica* (Takayanagi *et al.*, 1986; Nagashima, 1995). Although chloroplast-assigned sequences in the short-read ana­lysis were present before filtering and accounted for 20–50% of total reads in field samples, it was not possible to taxonomically assign them to specific red algal taxa based on sequence information.

In addition, *A.* (*Thiobacillus*) *ferrooxidans* is known to‍ ‍inhabit the crater lake, Lake Yugama, which is located at an altitude of 2,100 m, in close proximity to the Yukawa River (Takano *et al.*, 1997). In the present study, samples of detritus and river water contained representatives of the order *Acidithiobacillales*, including *A. ferrooxidans* and *Acidiphilium* sp. These bacteria are chemotrophic and capable of growth in environments with elevated concentrations of protons and heavy metal ions (Rawlings, 2005). The majority of the bacterial species identified in detritus and river water in the present study, including representatives of the Sva0485, *Tepidisphaerales*, and *Leptospirillum* taxonomic groups, have been detected in acid mine drainage ecosystems, which typically have elevated concentrations of sulfur-containing compounds (Chen *et al.*, 2016; Tan *et al.*, 2019). The habitats preferred by these bacteria are similar to that of the Yukawa River, which is characterized by elevated concentrations of sulfur-containing compounds and heavy metal ions (Kikawada *et al.*, 2014).

*Acidithiobacillales* are capable of obtaining energy from the oxidation of sulfur and the reduction of inorganic sulfur compounds. *A. ferrooxidans* has been shown to proliferate by oxidizing sulfur or Fe(II) and also under conditions of high acidity (Valdés *et al.*, 2008; Quatrini *et al.*, 2009). A previous study found that iron oxidation and CO_2_ fixation were facilitated in a co-culture of *A. ferrooxidans* and *Acidiphilium* species (Liu *et al.*, 2011), indicating that these‍ ‍species provide a carbon source for the larvae of *Polypedilum* sp. Therefore, members of this microbiome, as chemolithoautotrophs and/or photoautotrophic primary producers, are presumed to sustain the growth of acid-tolerant *Polypedilum* sp. in the Yukawa River; however, further geochemical ana­lyses are required to confirm this possibility.

The site for sampling Yukawa River water and detritus on the riverbed was limited to the habitat of acid-tolerant *Polypedilum* sp. larvae. Therefore, this approach did not allow for a comprehensive survey of the Yukawa River microbiome. Instead, the goal of the present study was to examine the microbiome of acid-tolerant chironomids in their natural habitat. The river water sampling point was restricted to the habitat of chironomid larvae to serve as a control in the ana­lysis of the chironomid microbiome, which was exami­ned at the individual specimen level to reveal intra-individual variations and putative obligatory relationships.

### The microbiome of acid-tolerant larvae

Analyses of alpha and beta diversities indicated that bacteria were not the sole source of nutrition for *Polypedilum* sp. larvae. The alpha diversity of larval samples was lower than those of detritus, river water, and egg masses, indicating that specific strains of bacteria were enriched within larvae. Additionally, the beta diversity ana­lysis revealed distinct phylogenetic dissimilarities between the bacterial communities associated with the larvae and those associated with the other sample types. The predominant bacterial groups present in larvae at their habitat were *Acidithiobacillales*, *Eubacteriales*, and *Caryophanales*. *Eubacteriales* and *Caryophanales* were more abundant in larvae than in the other samples. A comparison of the full-length 16S rRNA gene sequences of the larval microbiome revealed the presence of taxa exhibiting <98.65% similarity to known species, which indicates that this value represents a threshold for distinguishing microbial species (Kim *et al.*, 2014). Therefore, *Polypedilum* sp. larvae appear to harbor unique, as yet unknown, bacterial species.

Among the unique bacteria identified, OTU_*Bacillaceae*_Yukawa was the most closely related to *Oikeobacillus pervagus*, as known as *Bacillus pervagus* (Narsing Rao *et al.*, 2023), which exhibits growth in the pH range of 5 to 9.5 (Kosowski *et al.*, 2014). OTU_*Lachnospiraceae*_Yukawa is closely related to *Anaeropeptidivorans aminofermentans*, which shows growth in the pH range of 7.5 to 8.5 (Köller *et al.*, 2022). Acidic conditions appear to be an unfavorable environment for the growth of these bacteria. The prevalence of OTU_*Bacillaceae*_Yukawa and OTU_*Lachnospiraceae*_Yukawa in the larval microbial community suggests that the inside of larvae provide an environmental niche that is more conducive to growth than the external acidic environment.

A comparison of the microbiome of acid-tolerant *Polypedilum* sp. larvae collected in their natural habitat with that of larvae reared in the laboratory for more than 4 months revealed that OTU_*Acidithiobacillus* sp._Yukawa and OTU_*Lachnospiraceae*_Yukawa were not present in the microbiome of laboratory-reared larvae, whereas OTU_*Bacillaceae*_Yukawa remained detectable (Fig. 6, Table S4 and S5). Since the life cycle of acid-tolerant *Polypedilum* sp. is approximately 1 month, the rearing period in the laboratory corresponded to at least 4 generations after collection from the field. OTU_*Bacillaceae*_Yukawa was not detected in either the river water of the habitat or the rearing water in our laboratory. This absence suggests that OTU_*Bacillaceae*_Yukawa is vertically transmitted across generations of acid-tolerant *Polypedilum* sp. and plays an essential role in the survival of the chironomid. In some insect species, the vertical transmission of symbiotic bacteria is mediated by the materials that surround the eggs (Nyholm, 2020). In this acid-tolerant chironomid, egg masses, comprising multiple eggs embedded in a gelatinous matrix, are laid as clusters on rocks just above the surface of the stream (Fig. 1C). Although sequences for the order *Caryophanales* were detected in the egg mass sample (Fig. S4), no 16S rRNA gene sequences corresponding to OTU_*Bacillaceae*_Yukawa were found. To clarify the mechanism of vertical transmission, further studies focusing on the behavioral ecology of the acid-tolerant *Polypedilum* sp. and comprehensive microbiome ana­lyses are warranted.

In addition, multiple OTUs belonging to *Mycobacteriales* were detected in the microbiome of larvae reared under laboratory conditions. This suggests that the presence of bacteria belonging to *Mycobacteriales*, irrespective of specific species, plays an important role in the survival of *Polypedilum* sp. larvae. However, OTU_*Acidithiobacillus* sp._Yukawa, which disappeared in laboratory-reared larvae, may be continuously taken up from the environment in the natural habitat, where this OTU constitutes a major taxon.

The addition of nutrient-rich fish food to rear acid-tolerant chironomid larvae in the laboratory may suppress the growth of chemolithoautotrophic bacteria, such as *Acidithiobacillales*, which are adapted to the oligotrophic environment of the acidic Yukawa River. In contrast, heterotrophic bacteria are able to proliferate in the presence of fish food, which eventually results in an increase in the diversity of laboratory rearing water (Fig. 6A and B, Tables S4 and S5). Unclassified_OTU-1 was detected in both river and rearing water. Therefore, a detailed ana­lysis, including species identification, is required.

### Presumed metabolic relationship between chironomids and the microbiome

The results of the PICRUSt2 ana­lysis suggested that 14 metabolic pathways were enriched in the microbiome of acid-tolerant larvae in their natural habitat (Fig. 5). This ana­lysis predicted that the microbiome of acid-tolerant chironomid larvae may be characterized by the presence of the thiazole biosynthesis II (*Bacillus*) and L-lysine biosynthesis II pathways. These pathways may provide essential nutrients for the insect; however, it is unclear whether they contribute to the acid tolerance of the host.

Various limitations affect the prediction of pathways using PiCRUSt2 (https://github.com/picrust/picrust2/wiki/Key-Limitations). However, when considering only common pathways that are highly conserved among microorganisms, prediction accuracy markedly increases. The results of this ana­lysis provide a basis for considering the functions of the microbiome in the larvae of acid-tolerant chironomids. The next step is to investigate the validity of the preliminary hypotheses derived using PiCRUSt2. The expected metabolic pathways of the larval microbiome, which includes unidentified bacterial species, and their physiological contribution to the host need to be fully elucidated. Shotgun sequencing to obtain the complete DNA sequences of microbial communities may allow for differences in their characteristic metabolic systems to be exami­ned with greater precision.

### Acid-sensitive bacteria hide within acid-tolerant larvae

Using the BacDive database (https://bacdive.dsmz.de), we found that the pH range for the growth of bacteria closely related to OTU_*Bacillaceae*_Yukawa, as shown in Table 1, was 7–10 for *Pradoshia eiseniae* and 5–9 for *O. pervagus*. Assuming growth characteristics similar to those of these related species, OTU_*Bacillaceae*_Yukawa may be predicted to exhibit optimal growth in the neutral or weakly alkaline pH range. The heterogeneity observed in the bacterial phenotype of the larval microbiome suggests the tissue-specific localization of particular bacterial species according to the local pH. In the larvae of the acid-tolerant chironomid *Polypedilum* sp., OTU_*Bacillaceae*_Yukawa was only identified in segments 4–9, which roughly correspond to the larval midgut; OTU_*Bacillaceae*_Yukawa was not detected in segments 1–3 or 10–12, which are in close proximity to the mouth and anus, both of which open to the acidic external environment (Fig. 6C and S13). Although the pH in the gut of dipterans is predominantly neutral to alkaline, a limited region of the midgut is highly acidic, with a pH of 2, analogous to the vertebrate stomach (Dadd, 1975; Overend *et al.*, 2016). A previous study on two chironomid species reported a nearly neutral pH in the overall midgut (Frouz *et al.*, 2007).

In conclusion, the present study provides preliminary insights into the bacterial communities associated with the acid-tolerant chironomid *Polypedilum* sp. and its habitat. To fully elucidate the biological relationship between the acid tolerance of *Polypedilum* sp. and these bacteria, it will be necessary to investigate the biochemical, geochemical, and physiological properties of bacteria in the microbiome and conduct comprehensive ana­lyses of both microbial and host genomic sequences (*i.e.*, hologenomics).

### Data accessibility

The raw data and codes used in 16S rRNA gene sequence ana­lyses in this study are available in the GitHub repository (https://github.com/Kikawada-Lab-UT-NARO/Microbiota_Acid_Chironimid). Sequence data have been deposited in the DDBJ/NCBI/ENA BioProject database under accession number PRJDB18726. Of the six full-length 16S rRNA gene sequences listed in Table 1, four have been successfully deposited in the DDBJ/NCBI/ENA database under accession numbers PV500974–PV500977. All sequence information, including that not accepted for deposition, is available in the above-mentioned GitHub repository.

## Citation

Nakanishi, E., Cornette, R., Shimura, S., and Kikawada, T. (2025) Microbiome Associated with *Polypedilum* sp. (Diptera; Chironomidae), a Midge Adapted to an Extremely Acidic Environment. *Microbes Environ ***40**: ME24090.

https://doi.org/10.1264/jsme2.ME24090

## Supplementary Material

Supplementary Material

## Figures and Tables

**Table 1.** Overview and taxonomic classification of most abundant 16S rRNA gene sequences assembled with NanoCLUST

Cluster	Length (bp)	BLASTn			Phylogenetic ana­lysis			Reliable taxonomic group		Provisional name based on classification	Accession no.
		Best hit	Identity (%)		Closest species	Identity (%)		Family	Genus		
OTU_1	1,476	*Acidithiobacillus ferrooxidans*	99.39		*Acidithiobacillus ferrooxidans* strain ATCC 23270 (NR_074193.1)	99.39		*Acidithiobacillaceae*	*Acidithiobacillus*	OTU_*Acidithiobacillus ferrooxidans*_Yukawa	PV500974
OTU_2	1,474	*Acidithiobacillus caldus*	96.24		*Acidithiobacillus caldus* strain KU (NR_026517.1)	96.24		*Acidithiobacillaceae*	*Acidithiobacillus*	OTU_*Acidithiobacillus* sp._Yukawa	PV500975
OTU_3	1,491	*Pradoshia eiseniae*	92.73		*Oikeobacillus pervagus* strain 8-4-E12 (NR_125564.1)	89.12		*Bacillaceae*	—	OTU_*Bacillaceae*_Yukawa	PV500976
OTU_4	1,458	*Anaeropeptidivorans aminofermentans*	89.56		*Anaeropeptidivorans aminofermentans* strain M3/9 (NR_189185.1)	89.56		*Lachnospiraceae*	—	OTU_*Lachnospiraceae*_Yukawa	PV500977
OTU_5	1,445	*Corynebacterium dentalis*	72.32		—	—		—	—	Unclassified_OTU-1	
OTU_6	1,473	*Ehrlichia chaffeensis*	69.31		—	—		—	—	Unclassified_OTU-2	

Note: “Length” indicates the assembled sequence length. “Identity (%)” represents sequence similarity to the closest match in the database. Taxonomic assignments were made according to identity thresholds proposed by Hackmann (2025): ≥97.2% for species, 90.1–99.0% for genus, 80.1–94.1% for family, and <80% for unidentified bacteria or others.
