# Supplementary material for: Microbiome Associated with Polypedilum sp. (Diptera; Chironomidae), a Midge Adapted to an Extremely Acidic Environment
Source: Microbes Environ. 2025 Jun 21;40(2):ME24090. doi: 10.1264/jsme2.ME24090 (PMC12213058; doi:10.1264/jsme2.ME24090)
Supplement: Supplementary file 1 — Supplementary Material [file 40_24090_s1.pdf]

Supplementary materials

Microbiome associated with *Polypedilum* sp. (Diptera; Chironomidae), a midge adapted to an extremely acidic environment

Eita Nakanishi[1], Richard Cornette[2], Sachiko Shimura[2], Takahiro Kikawada[1,2*]

1. Department of Integrated Biosciences, Graduate School of Frontier Sciences, 277-8562, The University of Tokyo, Kashiwa, Chiba, Japan
2. Institute of Agrobiological Sciences, National Agriculture and Food Research Organization (NARO), 305-0851, Tsukuba, Ibaraki, Japan

Supplementary figures: Fig. S1–S13

Supplementary tables: Table S1–S5

Movie file:

https://doi.org/10.6084/m9.figshare.27237672.v1
Habitat of *Polypedilum* sp. larvae: Yukawa River (Kusatsu, Gunma Pre. Japan).
Greenish detritus is visible on the riverbed rocks, and small larvae can be seen swimming around the debris.

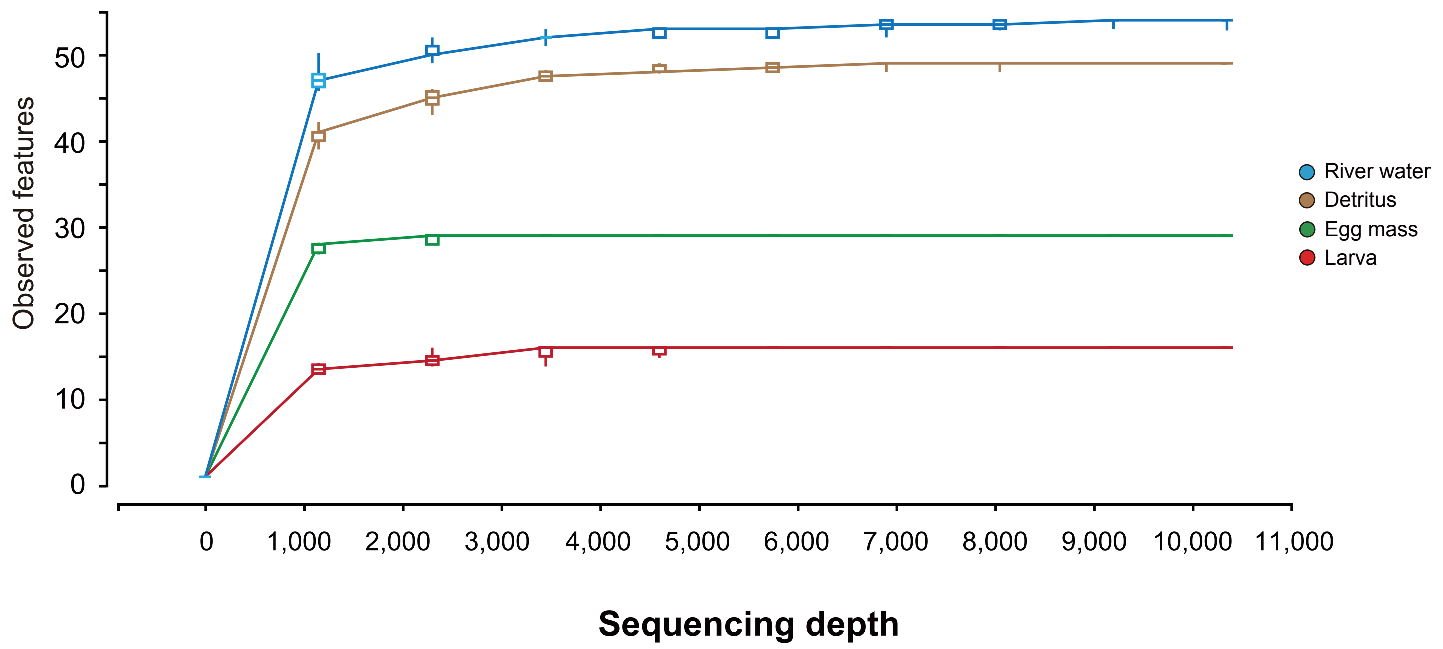

Fig. S1. Alpha rarefaction curves for river water, detritus, egg masses, and larval samples.

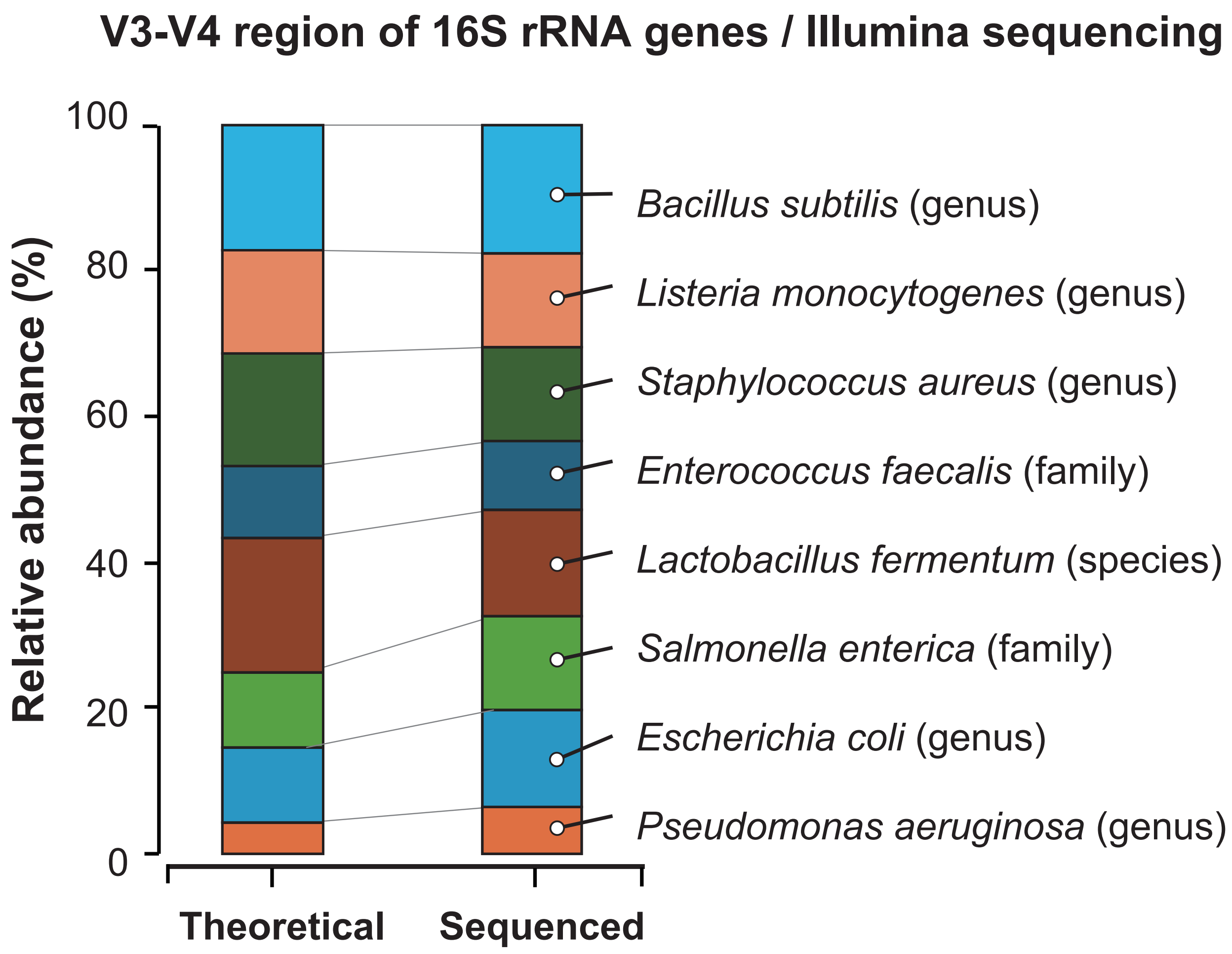

Fig. S2. The reliability of the workflow from library preparation to 16S rRNA sequencing analysis. Comparison of the composition profile between theoretical and actual sequenced samples of the mock community was illustrated. Parentheses indicate the taxonomy level given by taxonomic annotation using SILVA database.

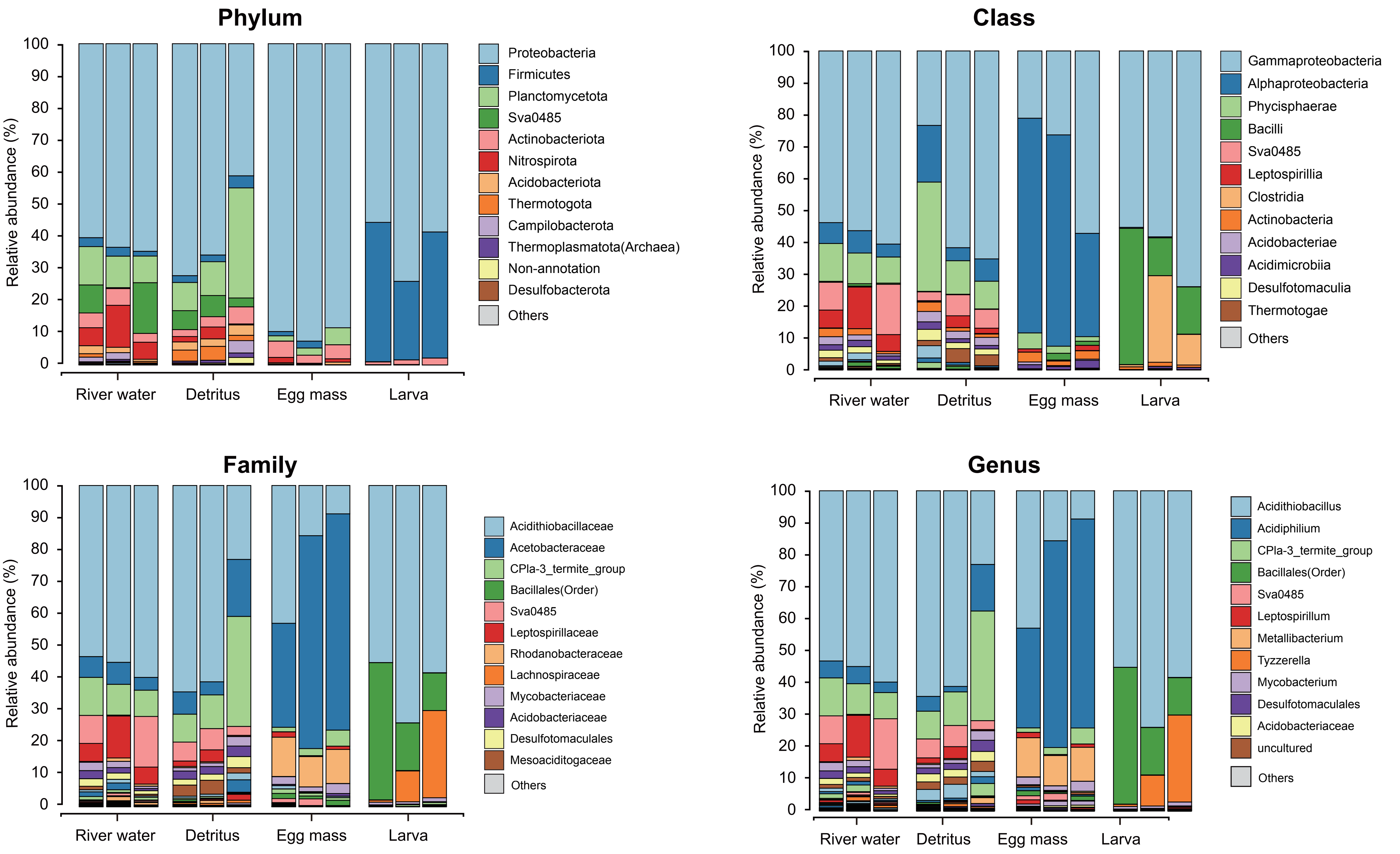

Fig. S3. Microbiome composition of larvae, eggs, and environmental samples.

Taxonomy bar plot for water, detritus, eggs, and larvae samples (n=3) at the order level. Y-axis represents relative abundance. Each color represents a taxonomic annotation, shown at different taxon levels.

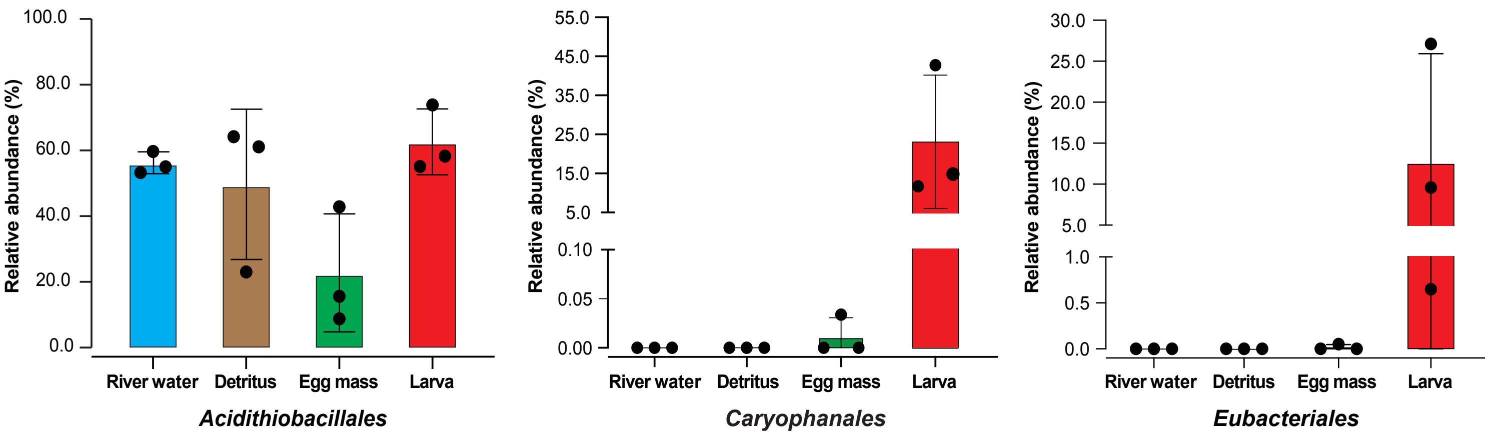

Fig. S4. Relative abundance of three specific order in the microbiomes of river water, detritus, egg mass, and larval samples.

The data were visualized using GraphPad Prism 10 software (GraphPad, San Diego, CA, USA), with means and standard deviations shown.

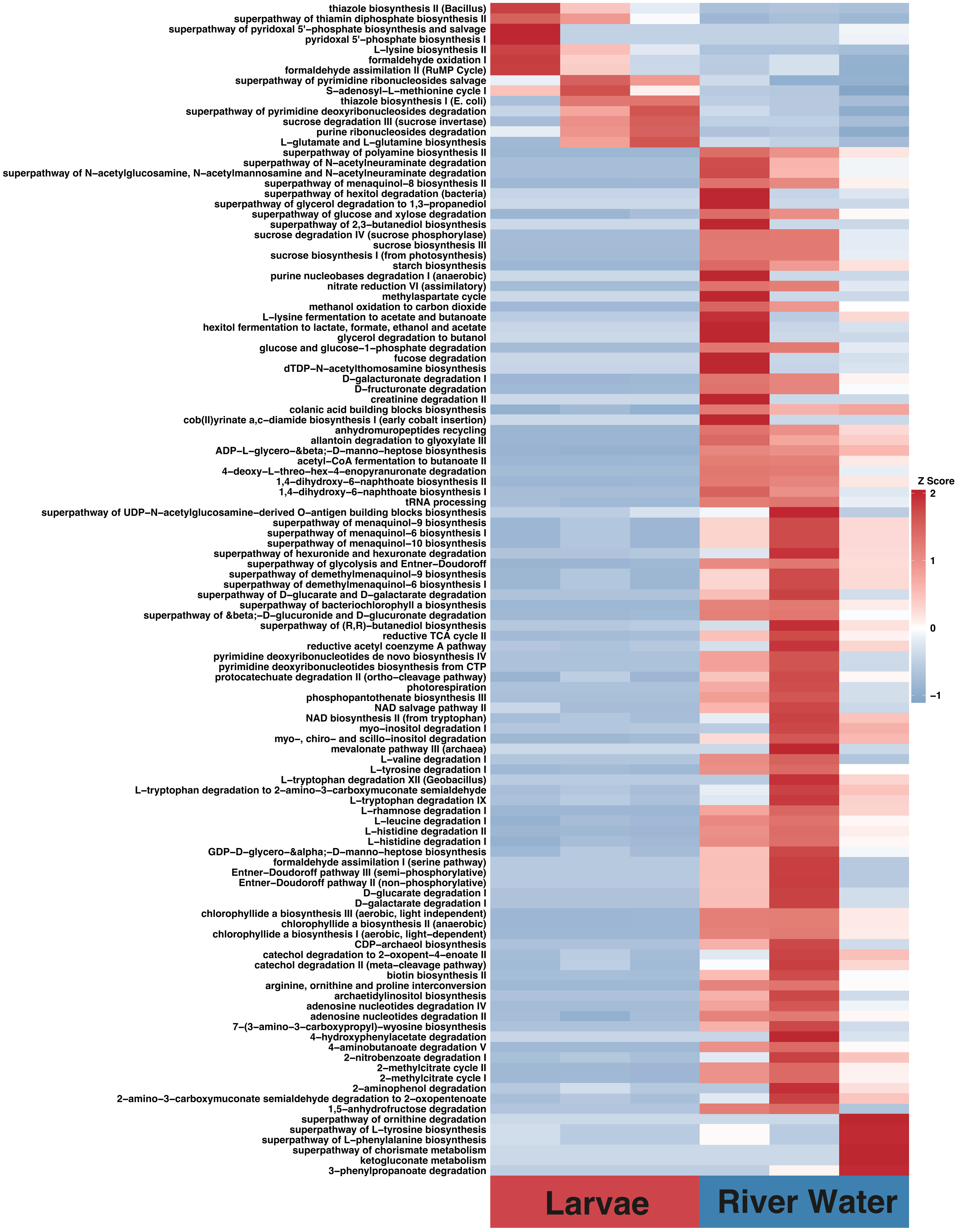

Fig. S5. Pathway features related to the larval microbiome using PICRUSt2 analysis.

As shown in the heat map, the abundance of 114 pathways in the microbiomes was predicted.

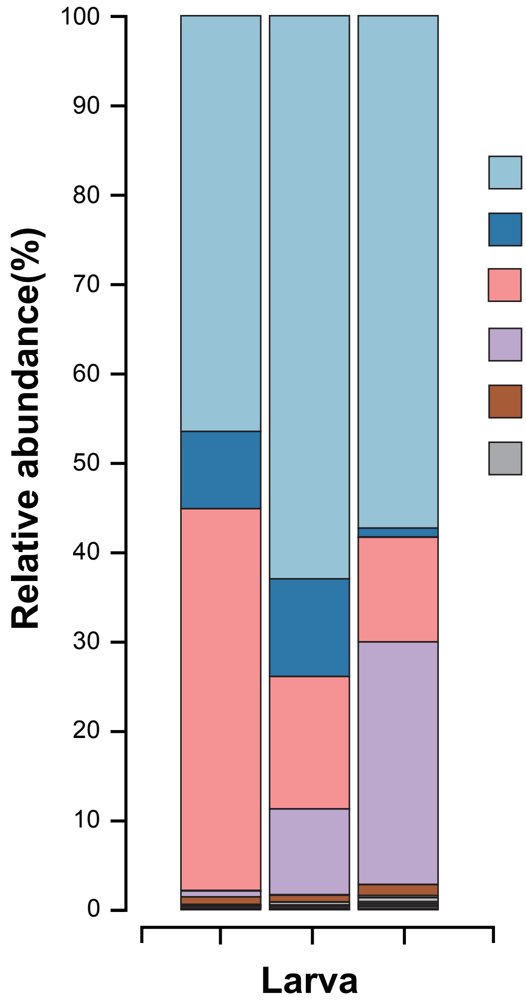

Taxonomy annotation			
Order	Family	Genus	Species
Acidithiobacillales	*Acidithiobacillaceae*	*Acidithiobacillus*	*Acidithiobacillus sp.*
Acidithiobacillales	*Acidithiobacillaceae*	*Acidithiobacillus*	*Acidithiobacillus ferrooxidans*
Caryophanales	-	-	-
Lachnospirales	*Lachnospiraceae*	*Clostridium*	-
Corynebacteriales	*Corynebacteriaceae*	*Mycobacterium*	-
Others			

Fig. S6. Annotation of larval samples using short-read sequening for V3-V4 region of 16S rRNA genes. The Y-axis indicates relative abundance of the taxa indicated in the key. The annotations range from order to species level. The ASV annotated as belonging to genus *Clostridium* was identified as *Anaeropeptidivorans* sp. when the full-length 16S rRNA gene sequence was used. The two genera belong to the same *Lachnospiraceae* family, and thus it is thought that the annotation is based on homology and is subject to fluctuation.

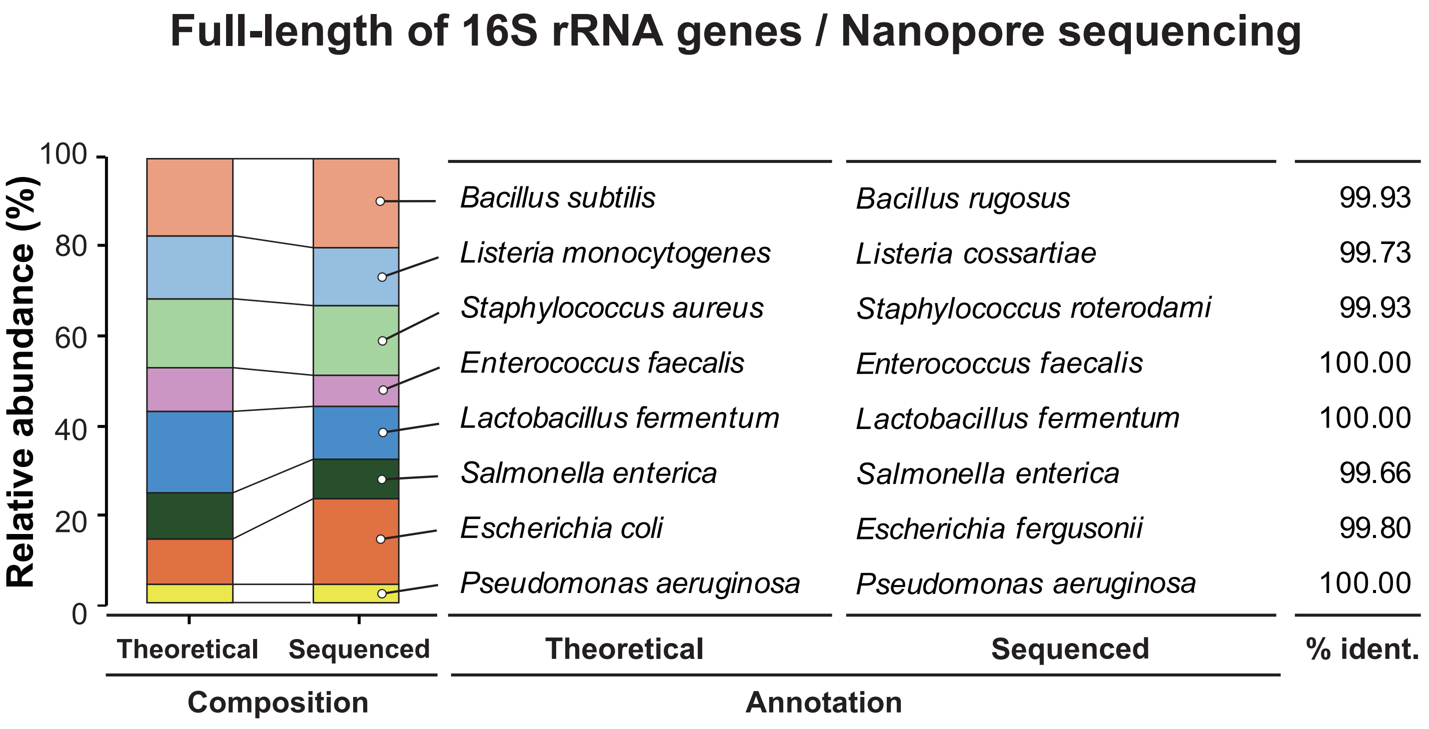

Fig. S7. Reliability of the workflow from library preparation to full-length 16S rRNA sequencing analysis.

The comparison of the compositional profiles between the theoretical values of the mock community and the actual sequence samples is shown. The similarity of the sequences is indicated by the identity with the NCBI 16S database.

Relative abundance

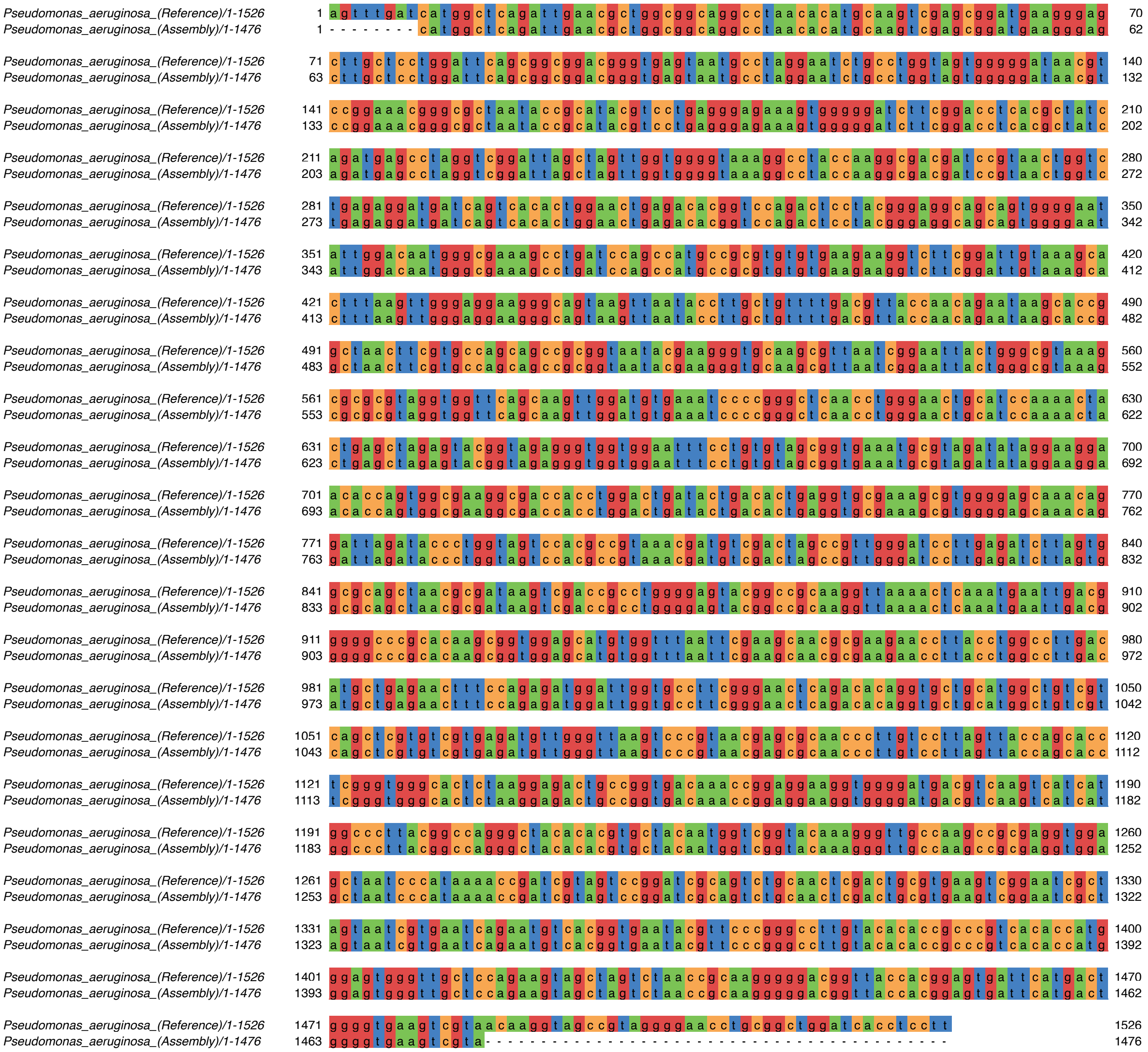

Fig. S8. Accuracy of the full-length sequence analysis of 16S rRNA genes using the nanopore sequencer. The identity of the sequenced data (Fig. S6) was determined through alignment analysis. For example, the predicted data (top) and actual sequence read assembly (bottom) for the full-length 16S rRNA gene of *Pseudomonas aeruginosa* in the mock community exhibited 100% matching. These results confirm that long-read sequencing and assembly were successfully and accurately executed using the nanopore sequencer.

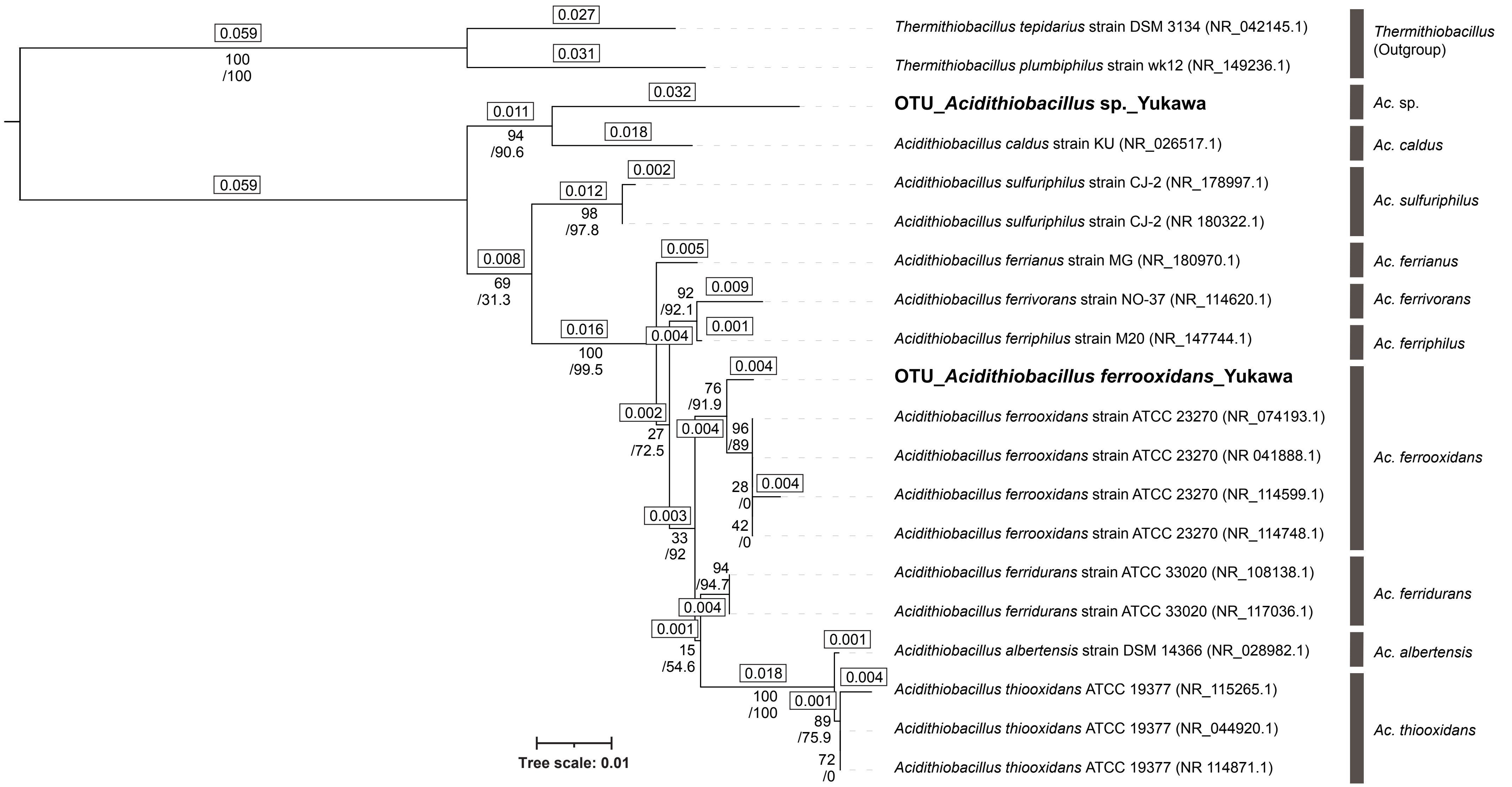

Fig. S9. Phylogenetic tree of *Acidithiobacillales* containing OTU_*Acidithiobacillus ferrooxidans*_Yukawa and OTU_*Acidithiobacillus* sp._Yukawa. The values not enclosed in squares on each branch are ultrafast bootstrap values (top) and SH-aLRT test values (bottom). A high confidence level is associated with a clade if it has SH-aLRT >= 80% and UFboot >= 95%. Branch lengths surrounded by squares below 0.000 are not indicated.

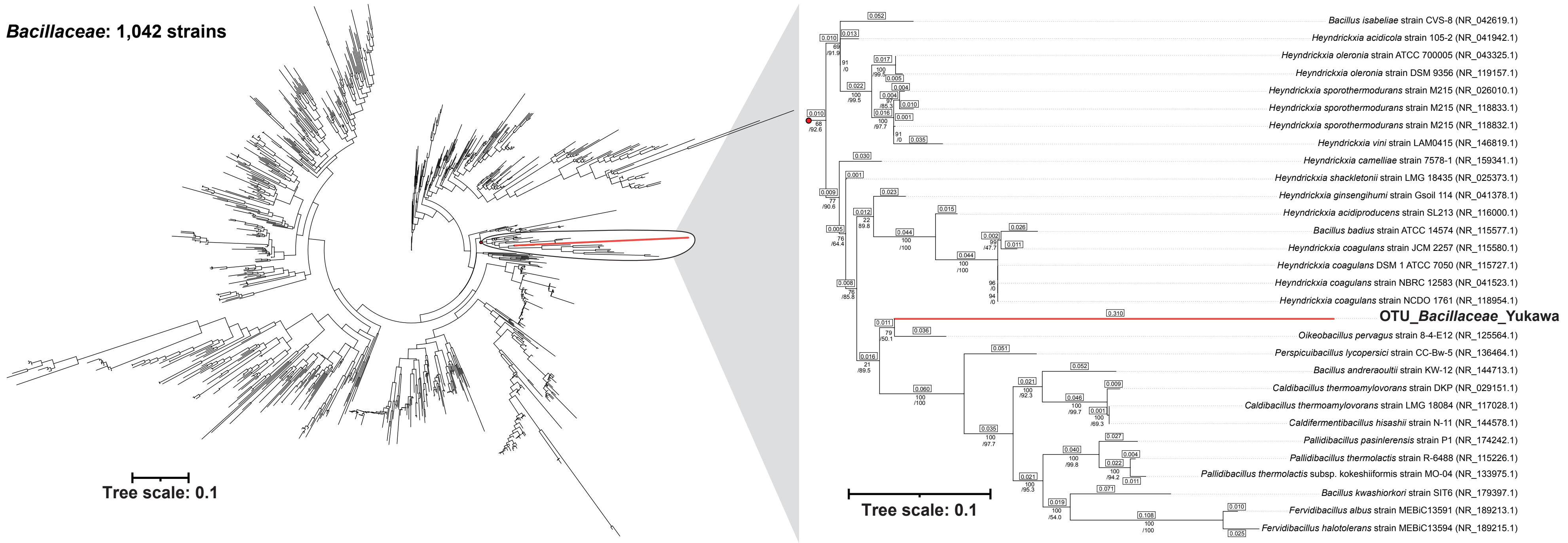

Fig. S10. Phylogenetic trees of OTU_*Bacillaceae*_Yukawa enriched in larvae compared to other samples.

The phylogenetic tree of the *Bacillaceae* family containing 1,042 strains (Left panel: Landscape, Right panel: focus). In right panel, the values not enclosed in squares on the branch are ultrafast bootstrap values (top) and SH-aLRT test values (bottom). A high confidence level is associated with a clade if it has SH-aLRT >= 80% and UFboot >= 95%. Branch lengths surrounded by squares below 0.000 are not indicated.

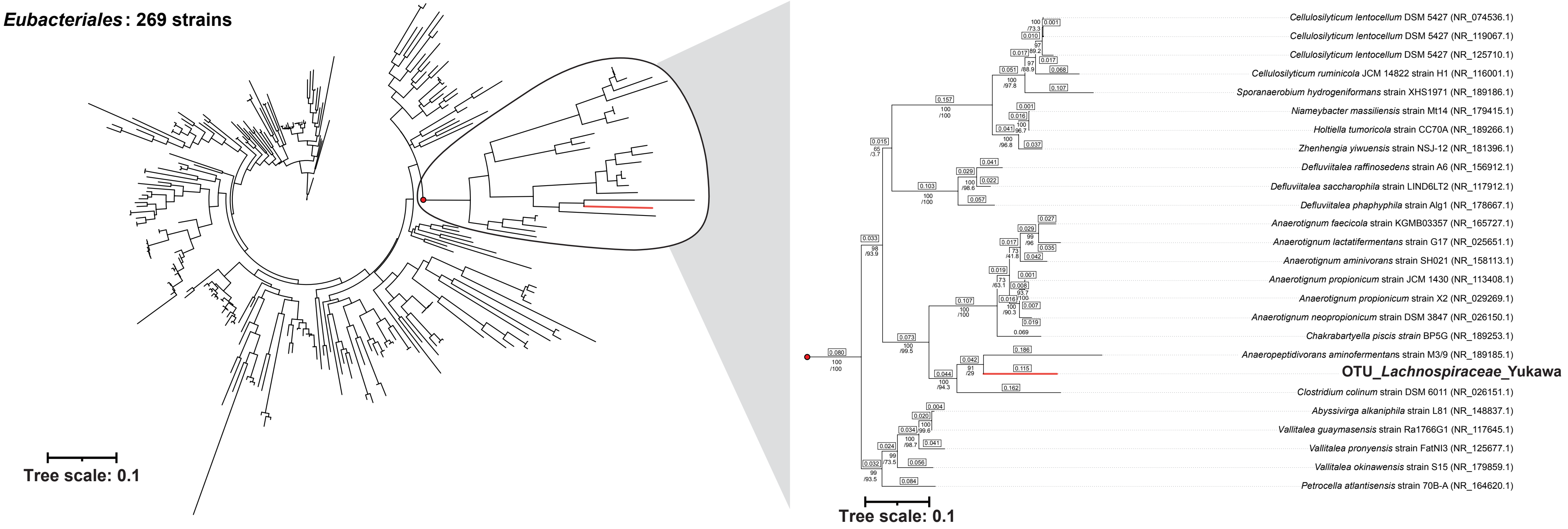

Fig. S11. Phylogenetic trees of OTU_*Lachnospiraceae*_Yukawa enriched in larvae compared to other samples.

The phylogenetic tree of the *Eubacteriales* order containing 269 strains (Left panel: Landscape, Right panel: focus). In right panel, the values not enclosed in squares on the branch are ultrafast bootstrap values (top) and SH-aLRT test values (bottom). A high confidence level is associated with a clade if it has SH-aLRT >= 80% and UFboot >= 95%. Branch lengths surrounded by squares below 0.000 are not indicated.

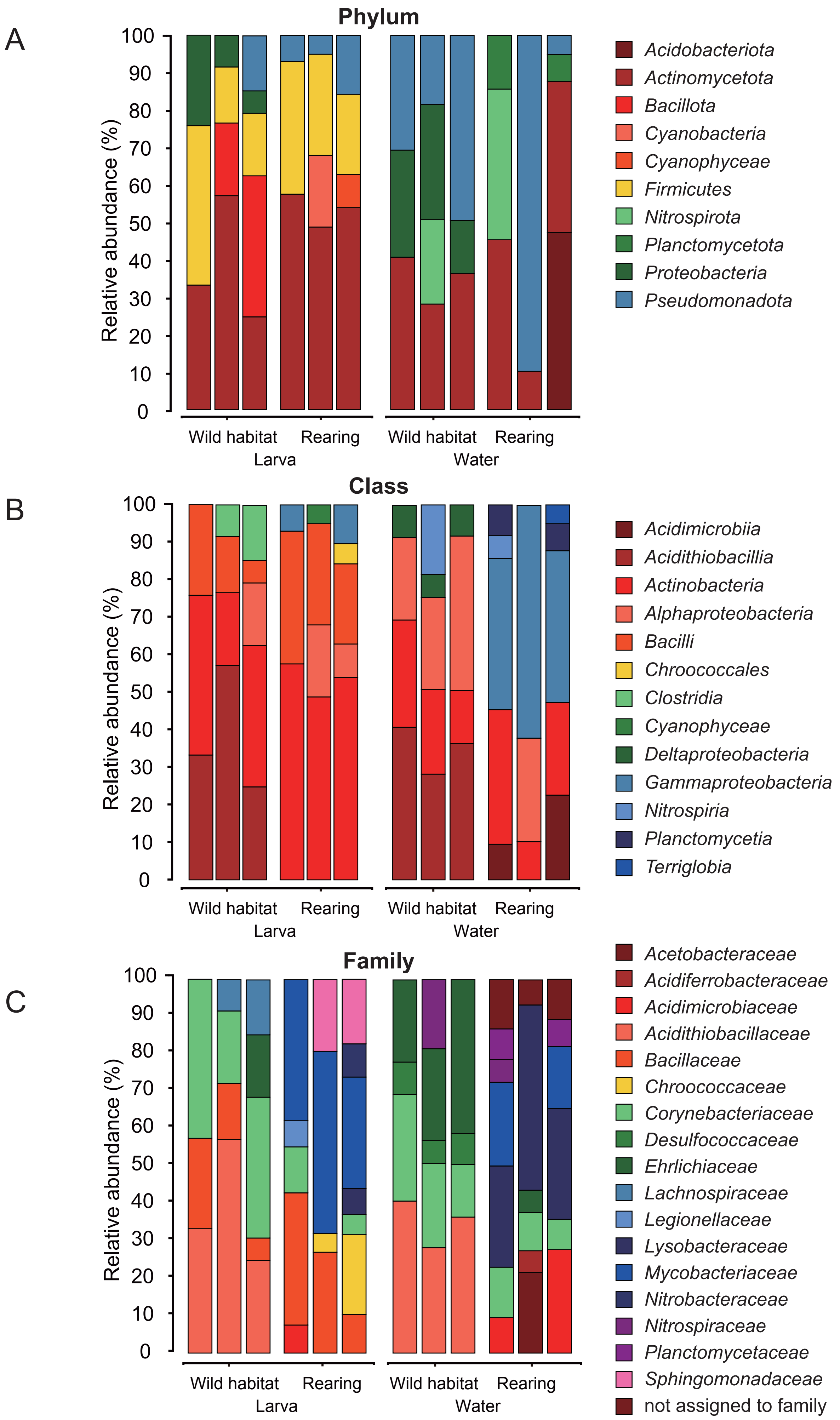

Fig. S12. Microbiome composition of larvae and rearing water under laboratory and wild conditions compared using long read analysis of full-length 16S rRNA genes, illustrated at different taxon levels.

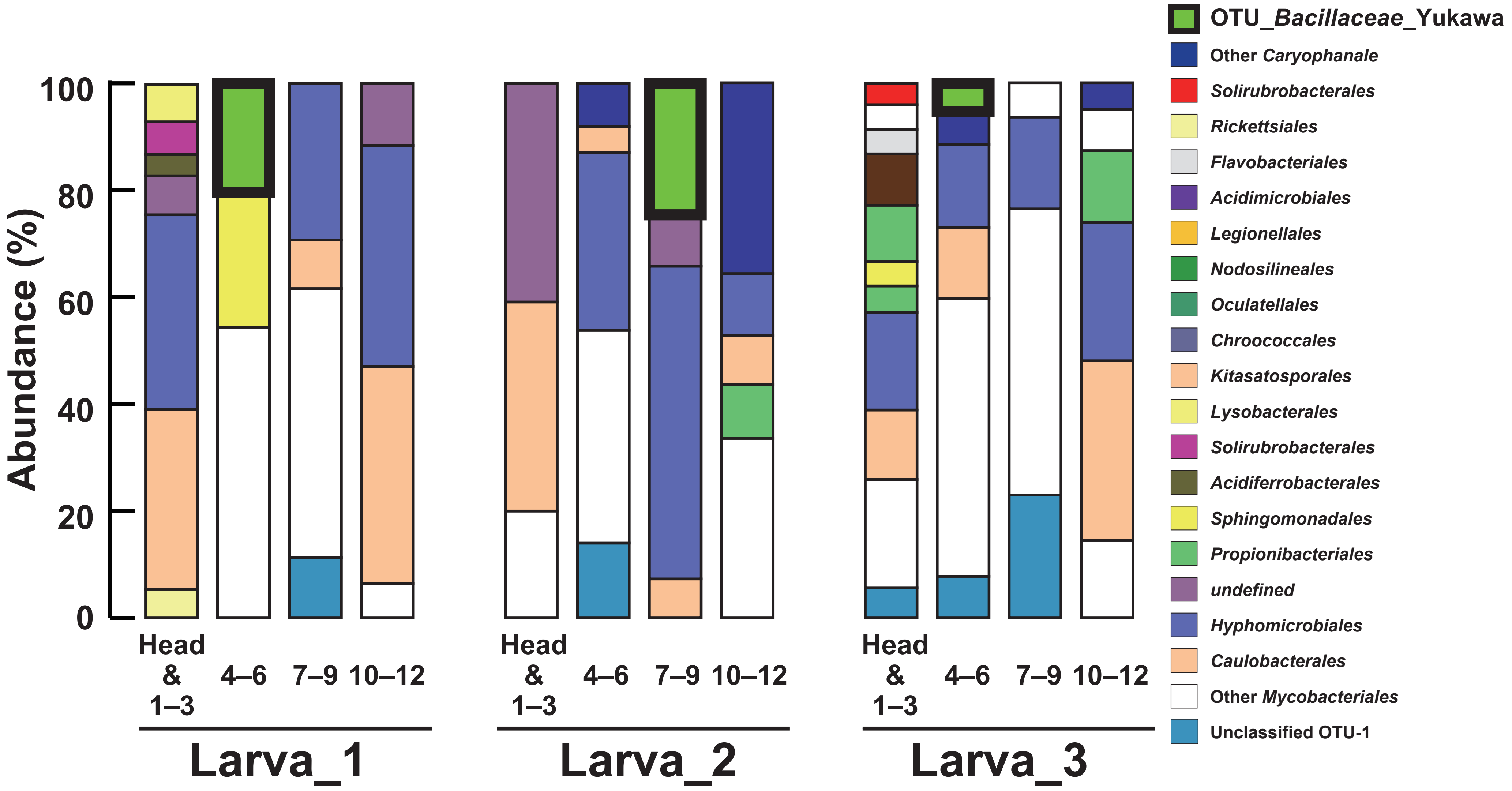

Fig. S13. Microbiome composition in different segments of the larval body.

Three individuals of the acid-tolerant chironomid larvae were sectioned through each of three body segments for 16S rRNA sequencing using the nanopore sequencer. OTU_*Bacillaceae*_Yukawa can be found in the midbody of the larvae, while OTU_*Corynebacteriaceae*_Yukawa was present in the midbody and the anterior region, including the mouth. Since OTU_*Corynebacteriaceae*_Yukawa exists in the rearing water, it should enter the bodies of the larvae when they feed.

Table S1. Number and percentage of reads in the short read sequencing of the 16S rRNA gene after being filtered using the DADA2 pipeline.

Sample name	Input (reads)	Filtered (reads)	Percentage of input passed filter (%)	Denoised (reads)	Merged (reads)	Percentage of input merged (%)	Non-chimeric (reads)	Percentage of input non-chimeric (%)	Chloroplast filtered (reads)
Egg_mass_1	52,921	23,344	44.11	23,209	22,893	43.26	22,596	42.70	17,748
Egg_mass_2	73,355	19,291	26.30	19,218	18,926	25.80	18,925	25.80	12,629
Egg_mass_3	41,106	18,267	44.44	18,204	18,021	43.84	18,020	43.84	11,554
Larva_1	35,673	26,978	75.63	26,823	26,704	74.86	26,547	74.42	15,271
Larva_2	38,657	23,145	59.87	22,997	22,870	59.16	22,628	58.54	18,212
Larva_3	38,376	20,753	54.08	20,643	20,581	53.63	20,517	53.46	10,349
Detritus_1	38,731	30,470	78.67	30,013	28,217	72.85	26,567	68.59	21,753
Detritus_2	40,687	31,929	78.47	31,431	29,667	72.92	27,786	68.29	22,841
Detritus_3	37,806	30,106	79.63	29,776	24,699	65.33	24,486	64.77	20,331
Water_1	37,143	30,449	81.98	29,959	28,542	76.84	27,624	74.37	16,050
Water_2	41,873	34,536	82.48	34,023	30,985	74.00	30,363	72.51	17,781
Water_3	36,222	30,309	83.68	29,904	28,908	79.81	28,166	77.76	12,441
Mock_community	37,288	31,211	83.70	31,094	29,857	80.07	27,374	73.41	22,505

Table S2. Number of nanopore sequencing reads for the full-length 16S rRNA gene.

Sample name	Raw reads		Filtered reads	
	number of sequences	average length (bp)	number of sequences	average length (bp)
Larva_1_wild_habitat	47,301	1,159.5	28,868	1,436.9
Larva_2_wild_habitat	32,762	1,189.9	19,778	1,445.4
Larva_3_wild_habitat	60,143	1,109.9	36,139	1,434.3
Larva_1_rearing	23,443	1,419.7	19,846	1,451.1
Larva_2_rearing	22,579	1,444.6	18,972	1,459.6
Larva_3_rearing	9,594	1,407.1	7,580	1,452.9
Water_1_wild_habitat	65,833	1,207.0	39,395	1,433.9
Water_2_wild_habitat	11,437	1,171.5	6,265	1,421.7
Water_3_wild_habitat	5,983	1,297.0	3,095	1,417.9
Water_1_rearing	7,597	1,273.6	5,605	1,442.5
Water_2_rearing	9,722	1,374.8	7,951	1,449.2
Water_3_rearing	9,921	1,309.8	7,932	1,449.0
Mock community	42,450	1,262.8	32,625	1,467.5
Larva_1_rearing_seg-1-3_w/_head	4,938	1,318.5	3,466	1422.6
Larva_2_rearing_seg-1-3_w/_head	3,160	1,487.3	1,541	1,546.7
Larva_3_rearing_seg-1-3_w/_head	4,663	1,327.9	3,475	1,447.1
Larva_1_rearing_seg-4-6	33,662	1,422.9	28,970	1,452.6
Larva_2_rearing_seg-4-6	20,718	1,403.4	16,520	1,453.4
Larva_3_rearing_seg-4-6	5,558	1,389.0	4,651	1,440.9
Larva_1_rearing_seg-7-9	9,699	1,379.5	7,983	1,442.6
Larva_2_rearing_seg-7-9	6,802	1,464.4	5,177	1,460.9
Larva_3_rearing_seg-7-9	15,724	1,394.0	13,713	1,441.5
Larva_1_rearing_seg-10-12	4,886	1,303.3	3,679	1,421.1
Larva_2_rearing_seg-10-12	3,678	1,394.3	3,198	1,441.2
Larva_3_rearing_seg-10-12	7,367	1,364.1	6,157	1,424.2

Note: Filtered reads indicate reads with a quality score of Q9 or higher and a sequence length of 1,000 bp or greater than 2,000 bp.

Table S3. Gaps and match rates for the full-length 16S rRNA gene in the sequenced mock community DNA.

Reference species	5'gap (bp)	3'gap (bp)	Sequence match rates other than 5' or 3' gaps (%)
Bacillus subtilis	20	48	100
Enterococcus faecalis	17	47	100
Salmonella enterica	11	43	100
Staphylococcus roterodami	20	46	100
Escherichia coli	18	44	100
Lactobacillus fermentum	12	41	100
Listeria monocytogenes	17	45	100
Pseudomonas aeruginosa	8	43	100

Table S4. Comparison of microbiome composition of the larvae between laboratory and wild conditions at OTU-level.

	Larva_wild_habitat_1	Larva_wild_habitat_2	Larva_wild_habitat_3	Larva_rearing_1	Larva_rearing_2	Larva_rearing_3
**OTU_*Bacillaceae*_Yukawa**	0.242	0.150	0.060	0.282	0.270	0.160
**OTU_*Acidithiobacillus ferrooxidans*_Yukawa**	0.055	0.085				
**OTU_*Acidithiobacillus* sp._Yukawa**	0.278	0.486	0.248			
**OTU_*Ehrlichiaceae*_Yukawa**			0.167			
**OTU_*Lachnospiraceae*_Yukawa**		0.084	0.147			
**OTU_*Aciditerrimonas* sp.**				0.075		
**OTU_*Bacillaceae*_2**						0.054
**OTU_*Bacillaceae*_3**				0.072		
**OTU_*Bradyrhizobium japonicum***						0.089
**OTU_*Chroococcaceae***					0.050	0.054
**OTU_*Legionellaceae***				0.070		
**OTU_*Metallibacterium* sp.**						0.103
**OTU_*Mycobacterium cookii***				0.153	0.239	0.233
**OTU_*Mycobacterium* sp._1**				0.140	0.249	0.173
**OTU_*Mycobacterium* sp._2**				0.085		0.065
**OTU_*Sphingomonadaceae***					0.192	
**Unclassified OTU-1**	0.425	0.194	0.377	0.123		0.070

Table S5. Comparison of microbiome composition of water between laboratory and wild conditions at OTU-level.

	Water_Wild_habitat_1	Water_Wild_habitat_2	Water_Wild_habitat_3	Water_rearing_1	Water_rearing_2	Water_rearing_3
**OTU_*Ehrlichiaceae*_Yukawa**	0.220	0.245	0.412			
**OTU_*Acidithiobacillus* sp._Yukawa**	0.203	0.160	0.154			
**OTU_*Acidithiobacillus ferrooxidans*_Yukawa**	0.205	0.123	0.210			
**OTU_*Desulfococcaceae*_Yukawa**	0.086	0.062	0.083			
**OTU_*Leptospirillum ferrooxidans*_Yukawa**		0.185				
**OTU_*Acidibacter* sp.**				0.132	0.067	0.108
**OTU_*Acidibrevibacterium fodinaquatile***					0.073	
**OTU_*Acidiferrobacteraceae***					0.058	
**OTU_*Acidimicrobiaceae*_1**				0.095		0.046
**OTU_*Acidimicrobiaceae*_2**						0.181
**OTU_*Acidiphilium multivorum***					0.143	
**OTU_*Ehrlichiaceae*_2**					0.060	
**OTU_*Granulicella* sp.**						0.050
**OTU_*Metallibacterium* sp.**				0.271	0.496	0.297
**OTU_*Mycobacterium cookii***				0.111		0.075
**OTU_*Mycobacterium* sp._1**				0.058		0.046
**OTU_*Mycobacterium* sp._2**				0.056		0.045
**OTU_*Nitrospiraceae***				0.061		
**OTU_*Planctomycetaceae***				0.082		0.072
**Unclassified OTU-1**	0.286	0.226	0.141	0.135	0.102	0.081
